# Chemical and isotopic composition of CO_2_-rich magnesium–sodium–bicarbonate–sulphate-type mineral waters from volcanoclastic aquifer in Rogaška Slatina, Slovenia

**DOI:** 10.1007/s10653-021-01062-2

**Published:** 2021-09-09

**Authors:** Nina Rman, Teodóra Szőcs, László Palcsu, Andrej Lapanje

**Affiliations:** 1 Geological Survey of Slovenia (GeoZS), Dimičeva ulica 14, 1000 Ljubljana, Slovenia; 2 Mining and Geological Survey of Hungary (MBFSZ), Columbus u. 17-23, 1145 Budapest, Hungary; 3 Isotope Climatology and Environmental Research Centre (ICER), Institute for Nuclear Research, Bem tér 18/c, 4026 Debrecen, Hungary

**Keywords:** Natural tracers, Carbon, Sulphur, Strontium and boron isotopes, Noble gases

## Abstract

Bottled natural mineral waters from an andesitic aquifer in Slovenia are enriched in magnesium (1.1 g/l), sulphate (2.2 g/l) and dissolved inorganic carbon (204 g/l). We analysed major ions, trace elements, tritium activity, ^14^C, *δ*^18^O_H2O_, *δ*^2^H_H2O,_ *δ*^13^C_DIC_*,* gas composition and noble gases in six wells. In addition, ^87^Sr/^/86^Sr, δ^34^S_SO4_ and *δ*^11^B were analysed here for the first time. Stable isotopes with *δ*^18^O = −11.97 to −10.30‰ and *δ*^2^H = −77.3 to −63.8 confirm meteoric origin. CO_2_ degassing is evident at three wells, causing the oxygen shift of about −1.3‰. Tritium activity was detectable only in the shallowest well, where the freshwater component was dated to the 1960s. *δ*^13^C_DIC_ in five waters is −1.78 to + 1.33‰, typical of carbonate dissolution. Radiocarbon is low, 1.03–5.16 pMC. Chemical correction with bicarbonate concentration and *δ*^13^C correction methods gave best mean residence times, slightly longer than previously published. Sulphate has *δ*^34^S 26.6–28.9‰ and *δ*^18^O 8.9–11.1‰ due to dissolution of evaporites in carbonate rocks. Boron at concentrations of 1.2–6.1 mg/l has two origins: *δ*^11^B = 11.3–16.4‰ from hydrothermal alteration and *δ*^11^B = 26.6–31.7‰ from carbonate dissolution. Strontium at concentrations of 0.5–22.0 mg/l has ^87^Sr/^/86^Sr, indicating three sources: 0.7106 for Miocene clastic rocks, 0.7082 for Triassic carbonates and 0.7070 for Lower Oligocene andesitic rocks. CO_2_ represents the majority of the dissolved (> 98.84 vol%) and separated gas (> 95.23 vol%). Methane is only found in two wells with a max. of 0.30 vol%. All waters show excess helium and 16–97% of mantle-derived helium. Since all show subsurface degassing, the paleo-infiltration temperature could not be calculated.

## Introduction

Mineral waters are usually related to specific geological conditions, a rather limited rate of recharge, and often an increased gas flux. There are not many groundwaters in the world that are as highly enriched in magnesium and sulphate as the mineral waters discussed in this paper.

Two recognized natural mineral water brands are currently produced in Rogaška Slatina, Slovenia, Tiha® (TDS = 0.5 g/l, Ca–Mg–HCO_3_) and Donat Mg® (TDS = 12–14 g/l, Mg–Na–HCO_3_–SO_4_/CO_2_ type). The water formerly referred to as Tempel (TDS = 3–4 g/l, e.g. Brenčič et al., 2010) is now a table water and is produced as a factory blend of both end-members. The Donat Mg® water has magnesium concentrations of up to 1.1 g/l and sulphate of 2.2 g/l and is produced from an andesitic aquifer. This distinguishes it from neighbouring groundwaters in the area, which are derived from either carbonate or clastic rocks, and also from other natural mineral waters known to the authors.

Bertoldi et al. (2011) studied 571 European mineral waters and found the highest magnesium values of 0.35 g/l and of sulphate 1.82 g/l, respectively. Birke et al. (2010a) surveyed 1785 European bottled waters and found magnesium only up to 0.25 g/l. Obviously, natural mineral waters from Rogaška Slatina were not included in these studies. In another paper, Birke et al. (2010b) listed that bottled waters in Germany have up to 0.242 g/l magnesium and 2.2 g/l sulphate. In Greece, magnesium reaches only 91 mg/l and sulphate 90 mg/l (Demetriades, 2010). In Italy, they are a maximum of 76 mg/l and 1.3 g/l (Dinelli et al., 2012), in Portugal only 37 and 110 mg/l (Lourenço et al., 2010) and in Chile 22 and 191.3 mg/l (Daniele et al., 2019). For Bulgaria, only magnesium is reported up to 41 mg/l (Lyubomirova et al., 2020). In France, the balneological effects of Hépar have been tested (Dupont et al., 2019), but it contains only 119 mg/l magnesium and 1.5 g/l sulphate.

In the Czech Republic, Magnesia contains only 156 mg/l magnesium and 11 mg/l sulphate, but two others are more interesting for our case (Hrkal et al., 2016). Šaratica evolved in very low permeability flysch with dolomitized limestones and dolomites, magnesites and gypsum lenses. It contains 790 mg/l magnesium and 7.7 g/l sulphate. Zaječicka Horka from siltstones has 5.1 g/l magnesium and 23.1 g/l sulphate. The enrichment in sulphates in both cases results from pyrite oxidation. Few CO_2_-rich mineral waters in Romania have magnesium up to 0.7 g/l and sulphate up to 4.2 g/l, mostly originating from halite domes and membrane filtration from clays (Kis et al., 2020). In Hungary, medicinal mineral waters of the magnesium–sulphate type have been known since the nineteenth century in South Buda, the present-day district of Budapest. These natural mineral waters are bottled under the brand names József Ferenc and János Hunyadi (Borszéki, 2013). Based on archival data from our previous sampling, labels and Borszéki (2013), the magnesium and sulphate contents of József Ferenc were 4.2 and 24 g/l, respectively, and have now decreased to about 1.4–2 g/l and about 13 g/l. The magnesium and sulphate concentrations of János Hunyadi have not changed significantly over the years, and vary between 2.2–2.8 and 18–20.3 g/l, respectively. Their source is a near-surface weathered Oligocene aquifer, where the high sulphate concentration is due to weathering of clays together with oxidation of pyrite, enhanced by oxygen-rich infiltrating precipitation (Gyalog et al., 2016).

This paper presents the most up-to-date information on the chemical and isotopic composition of the unique mineral waters from Rogaška Slatina. Previous works (Bräuer et al., 2016; Pezdič, 1997; Trček & Leis, 2017) interpreted their origin based on chemical composition, stable isotopes of oxygen, deuterium and carbon in the water, tritium activity, radiocarbon and noble gases. Our results allow re-evaluation of these conclusions and inclusion of additional data, for example, from the inactive K-2/75 well. We also supplemented the applied radiocarbon dating methods to account for mantle CO_2_ degassing and carbonate dissolution and pointed out the high uncertainty in assessment of mean residence time for such waters. Sulphur, strontium and boron isotopes in waters are extremely rarely analysed in Slovenia, and our examples also contribute to global knowledge. We aimed to improve the conceptual model about the origin and processes along the flow path of these mineral waters by distinguishing between three lithologies: carbonates in the recharge area, aquifers of andesitic rocks and siliciclastic aquitards. Our hypothesis is that the most prominent hydrogeochemical process is the dissolution of evaporites in carbonate rocks, which provides both high magnesium and sulphate content.

## Study area

### Geological settings

The geological features of the studies area are summarized in Trček and Leis (2017). Mineral water is stored in fractured Lower Oligocene volcanoclastic rocks. These are mostly tuff sandstone and breccia, and andesitic tuff and andesite of the Smrekovec Formation. Andesitic rocks have glassy matrix with plagioclases, pyrite, and nests of quartz and zeolites. Minerals show impacts of kaolinization, limonitization and calcification. In tuff, cement mostly comprises of clay and calcite, other minerals are as in andesite with addition of slate grains. The aquifer is rather narrow and extends in west–east direction; its width reduces from approximately 3000 m between Gabernik and Podplat to about 500 m at Rogaška Slatina. Its thickness is several hundred metres.

This aquifer is cut by two fault zones, the Donat in the north and the Šoštanj in the south. The sequence outcrops in two W–E directed patches. The one along the Donat Fault Zone is cut by the Labot Fault in the west. The second one outcrops south of the Šoštanj Fault Zone. The Šoštanj Fault Zone probably acts as a conduit for CO_2_ from deep sources (Bräuer et al., 2016).

The aquifer is covered by thick sequence of low permeable Neogene formations. The Upper Oligocene Pletovarje Formation starts with carbonate siltstone and sandstone which are followed by clayey carbonate siltstone. Above, the Upper Oligocene to Lower Miocene quartz sand and sandstone of the Govce Formation were deposited. Some layers are enriched in pyrite and marcasite.

Regional survey by Trček and co-workers (partly published in Trček & Leis, 2017) investigated the water flow dynamics, from precipitation and fresh groundwaters in Triassic carbonates and Miocene sandstone to the mineral water aquifer. Their findings resulted in a new hydrogeological model. The main recharge area is still presumed to be the carbonate complex of the Mts. Boč and Plešivec massif (with elevation up to 978 m a.s.l.) as presumed by Nosan (1973) and Pezdič (1997). However, its contact with low permeable Miocene clastic rocks is now assumed to be inclined and not thrusted. Therewith, the mountain can provide some, yet restricted recharge from the north.

### Hydrogeological settings

Mineral water springs between Rogaška Slatina, Gabernik and Kostrivnica emerge either along the north–south directed minor faults or at tuff outcrops. Several springs still exist but many have ceased over the years (reported already in Nosan, 1973). The mineral water aquifer in Lower Oligocene volcanoclastic rocks is poorly productive. Its maximum production rate is estimated to about 1.5 l/s per a well and the average production is only about 0.5 l/s. The water could be produced from five wells (Nosan, 1973, 1975; Trček & Leis, 2017): V-3/66-70 (for depths of interpreted wells, see Table 1), V-6/67 (265 m), K-2/75, K-2a/86 (534 m deep) and RgS-2/88. The latter borehole was unsuccessful; therefore, it was deepened in 1990 when the mineral water was successfully captured. In this paper, we name it RgS-2/88 and not RgS-2/88-90, as expected, because RgS-2/88 is used in official documents, e.g. concession decrees. V-3/66-70 is the only currently producing well. K-2/75 was producing till 1983 when the gas lift stopped due to production in V-3/66-70. Make-up well K-2a/86 was never exploited because it was hydraulically connected to both, and it was liquidated in 2019. It had casing issues, causing mixing of mineral water with water from the Pletovarje Formation clastic rocks, probably. Intrusion of a different water was noticed also in K-2/75 in 2015. Wells V-6/67 and RgS-2/88 are close-by. The first serves as a monitoring well while the second one is used for water drinking therapy at Medical Center Rogaška.

**Table 1:** General characteristics of interpreted wells. Production rates are taken from valid and past decrees on bottling water concessions mostly

Well name	Location	Predominant lithology	Depth (m)	Q_max_ (l/s)	Q_annual allowed_ (m^3^/y)
Kraljevi vrelec	Spodnja Kostrivnica	Ol, M clastic rocks	24	< 1	–
K-1/71	Zgornja Kostrivnica	Ol mixture of andesitic tuff and clastic rocks	170	< 0.1	–
K-2/75	Spodnja Kostrivnica	Ol andesitic tuff sandstone and breccia	546	4	–
V-3/66-70	Spodnji Gabernik		606	1.5	47,000*
RgS-2/88	Rogaška Slatina		277	0.5	12,600
G-10/95	Zgornji Gabernik	Ol siltstone and dolomitized andesitic tuff	603	0.4	12,500
Rt-1/92	Rogaška Slatina	T clastic rocks with dolomitized trachyte and diabase tuff	1700	4.25	69,379

*Quantity is granted jointly for V-3/66-70 and K-2a/86 well, the latter is not in operation any more. Ol = Oligocene, M = Miocene, T = Triassic

Several other mineral water aquifers are also tapped in the vicinity (Table 1). The least mineralized natural mineral water (brand Tiha) emerges from Triassic dolomite rocks at Boč Mt. Near by, wells G-10/95 and K-1/71 can produce middle mineralized water from a mixture of Oligocene tuff and siltstone close to the Boč Mt., named brand Tempel in the past. The 1.7 km deep well Rt-1/92 in Rogaška Slatina town produces thermomineral water from Triassic clastic rocks with dolomitized trachyte (Lapanje, 2006; Trček & Leis, 2017).

### Hydrogeochemical settings

Donat Mg mineral water brand has high concentrations of magnesium (1.1 g/l) and sulphate (2.2 g/l) which results in a distinctive Mg–Na–HCO_3_–SO_4_/CO_2_ water type (Lapanje, 2006). Total dissolved solids are between 12 and 14 g/l and gaseous CO_2_ in water is between 2 and 40 g/l (Nosan, 1973).

First isotopic studies (Pezdič, 1997) revealed its meteoric origin without any effect due to water–rock interactions or CO_2_ degassing. The average residence time was calculated to about 8000 years (or older) based on lower values of oxygen and hydrogen isotopes in comparison with fresh groundwater, indicating colder climate during infiltration, and radiocarbon dating applying chemical correction with bicarbonate concentration. Only the southern part of mineral water aquifer exhibited a constant isotopic composition at that time while the fresh groundwater in its northern part was dated to be younger than 30 years.

Investigation between 2007 and 2011 (Trček & Leis, 2017) found that mineral waters are enriched in volcanic CO_2_ and have high resemblance in organic compounds and microbiological parameters. The average residence time was determined to be from 3400 years (RgS-2/88) to 7200 years (V-3/66-70 and G-10/95) and to 14,000 years for thermomineral water (Rt-1/92). Microbial diversity of these waters was investigated also by Börger (2017).

Gases were first surveyed by Pezdič (1997). It was almost pure CO_2_ with 0.3% of nitrogen and oxygen and methane below 0.01%. Bräuer et al. (2016) investigated their origin using noble gases. They confirmed high purity of CO_2_ gas (99.7–99.9 vol%) and abundance of high fraction (> 75%) of mantle-derived helium, some geogenic argon and CO_2_ from lithospheric mantle. This is supported also by slightly modified volcanic/magmatic *δ*^13^C_CO2_, being −6.1‰ in RgS-2/88 and −4.9‰ in V-3/66-70 instead of typical −3.5‰. RgS-2/88 has predominately an air-saturated water component and shows He loss during the migration away from the magma degassing centre. V-3/66-70 shows more mantle-derived component and slightly less fractionation.

Thermomineral water from well Rt-1/92 with 55.4 °C has mineralization of 6 g/l and is of Na–HCO_3_–SO_4_/CO_2_ water type (Lapanje, 2006). Its mean residence time was estimated to 14,000 years (Trček & Leis, 2017).

## Methods

In this paper, we distinguish among terms mineral and natural mineral water. While the first describes waters with TDS above 1 g/l, the second one is used only if we want to emphasize recognized bottled water brands according to two European directives on natural mineral waters (Directive, 2009/54/EC and Directive, 2003/40/EC).

### Sampling procedure

Field work was performed on 6 and 7 September 2016 by the Accredited Water Sampling Group of the Mining and Geological Survey of Hungary (MBFSZ) with field support from Geological Survey of Slovenia (GeoZS). At that time, wells V-3/66-70 and RgS-2/88 were in constant production by gas lift. Well K-2/75 was airlifted 1 day before the sampling, while activation of K-2a/86 was unsuccessful. Consequently, we sampled Kraljevi vrelec instead. G-10/95 was activated and sampled as an exchange site instead of low-flow artesian well K-1/71. Only few parameters were determined for the latter. There are also two other difficulties encountered during the sampling: gas sampling at V-3/66-70 was possible only after the gas separator and not at the wellhead (Fig. 2), and free and dissolved gas samples at RgS-2/88 were not available to us.

**Fig. 1:**
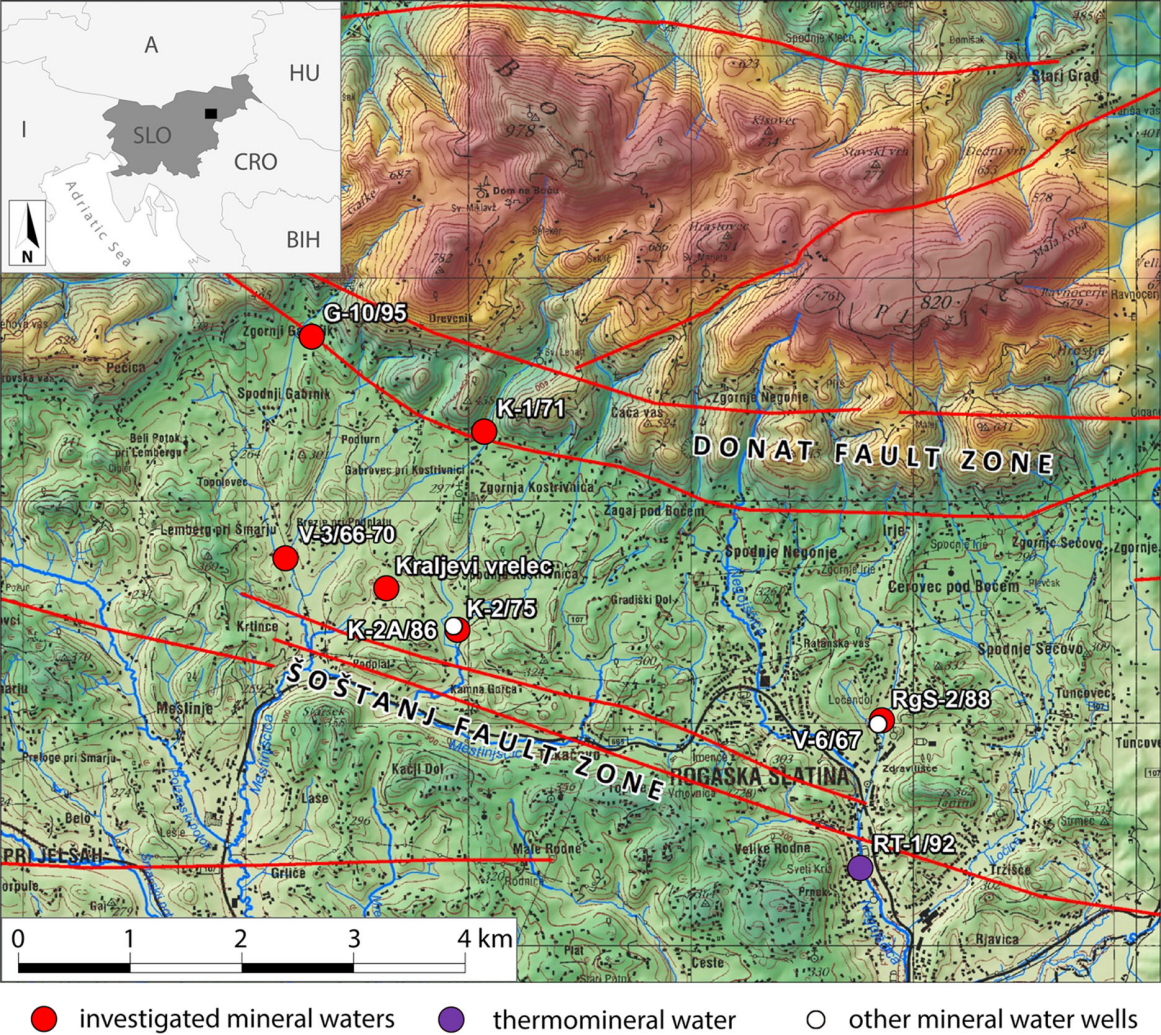
Investigated water wells in Rogaška Slatina and its surroundings with locations of major fault zones. Map: GURS: National topographic map at scale 50,000, 2006–2017

**Fig. 2:**
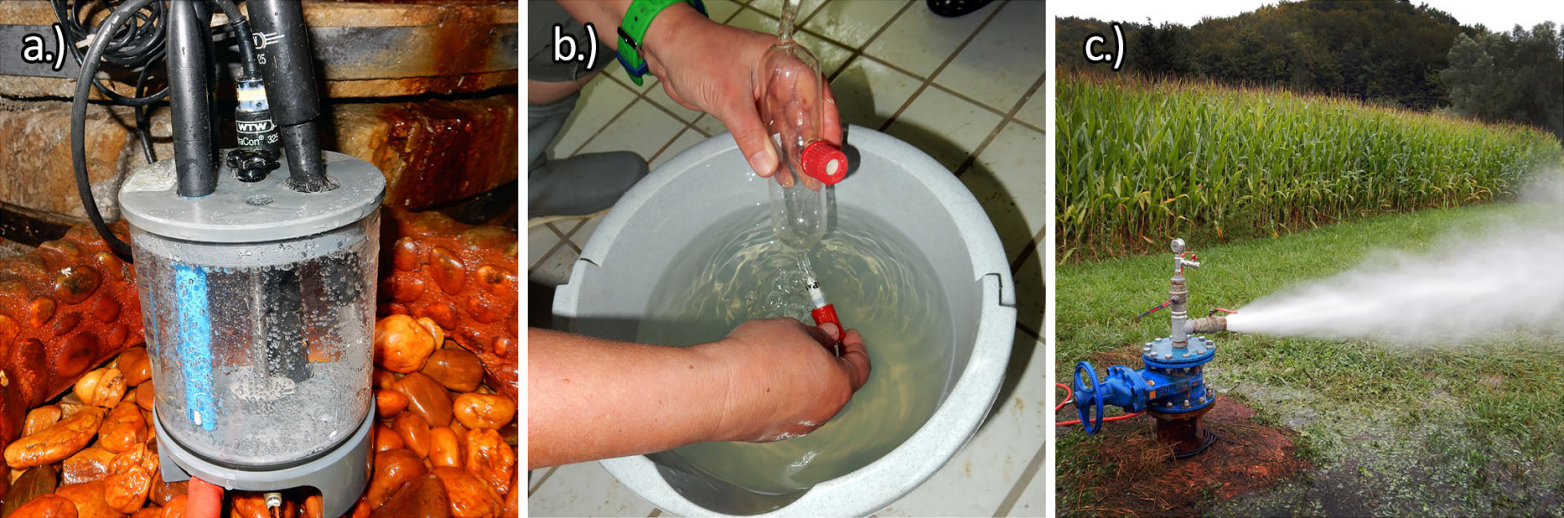
Example of sampling sites:** a** Kraljevi vrelec, **b** V-3/66-70 water and gas after the separator and **c** gas lift at K-2/75. All have lots of free gas and show iron scaling (notice orange precipitates (**a**, **c**) and brownish colour in the bucket (**b**))

Samples for alkalinity and electric conductivity measurements were collected in 0.5-l plastic bottles and filled completely, airtight and stored between 2 and 5 ºC. For cation and trace element determination 100 ml water was filtered through a 0.45 µm pore size membrane and preserved with 2 ml extra pure HNO_3_, while for nitrate, nitrite, chloride and sulphate determination a separate, also filtered, sample was collected in a 20 ml plastic bottle, closed airtight and stored between 2 and 5 ºC. Samples for ammonium determination were collected in plastic bottles adding 0.2 ml 1:1 diluted H_2_SO_4_ to 100 ml water. Bicarbonate was determined from samples collected in dark glass bottles adding 450 ml double distilled water to 50 ml groundwater sample and then stored between 2 and 5 ºC. Samples for COD measurements were collected in glass bottles adding 100 ml filtered water through a 0.45 µm pore size membrane and preserved with 1 ml 96 m/m% extra pure H_2_SO_4_. Samples for total (TOC) and dissolved (DOC) organic carbon and dissolved inorganic carbon (DIC) were filled airtight in two 500-ml dark glass bottles for each sample and stored between 2 and 5 ºC. Samples for TOC determination were collected in transparent glass bottles adding 10 ml 2 M HCl to 100 ml water and stored at 2–5 ºC.

Water samples for *δ*^18^O and *δ*^2^H analyses were collected in 50 mL HDPE bottles with no headspace. Collected samples were sealed with PARAFILM to prevent evaporation. Samples for tritium measurements were collected in 3 l plastic cans. Water and gas samples for noble gas analysis were taken into copper tubes closed by pinch-off clamps.

Samples for radiocarbon dating were collected in plastic vessels varying between 5 and 10 l, depending on the bicarbonate concentrations of the sampled groundwater determined based on field titration for alkalinity. The amount of collected water was calculated to ensure an approximately 6 g of carbon in the BaCO_3_ precipitation which was gained by adding carbonate free NaOH to increase the pH of the sample above 11 and then BaCl_2_ was added to the sample. A minimal contact with air was ensured during sampling. For *δ*^13^C_DIC_, *δ*^34^S and *δ*^18^O from sulphate measurements samples were collected in the same way as for the radiocarbon age determination, but in a separate 5 l plastic can.

Samples for ^87^Sr/^86^Sr and *δ*^11^B were filtrated through 0.45 and 0.20 μm nylon filters into a 60 ml acid leached LDPE bottles and packed individually, one bottle per zip-lock bag.

Samples for dissolved gas analysis were taken directly in two 0.75 l glass bottles closed with a rubber teat without gas bubbles, while separated gas was sampled with a ‘house made’ field gas separator and stored in a two-valve system glass tube. Samples for noble gas analysis were collected in two 20 ml copper tubes for each sample and closed tight under water and under pressure, respectively.

Well Rt-1/92 was sampled in November 2016 independently of this research within annual monitoring requirements for water concession (Hötzl & Rman, 2017). Sampling and chemical analyses were performed by accredited National Laboratory of Health, Environment and Food in Maribor, Slovenia, while other labs were the same as for presented research. Only samples for B and Sr isotopes were taken in September for sole purpose of this study.

### Analytical methods

Main and trace elements were determined at MBFSZ laboratory in Budapest. Na^+^, K^+^, Ca^2+^, Mg^2+^, Fe^2+^, Mn^2+^, PO_4_^3−^, SO_4_^2−^ and H_2_SiO_3_ were analysed by ICP-AES Jobin Yvon ULITIMA 2C, and anions Cl^−^, NO_2_^−^ and NO_3_^−^ by IC equipped with Waters 431 conductivity detector as well as with Perkin Elmer Series 200 UV/VIS detector. NH_4_^+^ and F^−^ were determined by photometry; the first with Nanocolor 400 D photometer, while the second with Hach DR 3900 photometer. HCO_3_^−^, CO_3_^2−^ and OH^−^ were calculated based on titration of alkalinity. COD was determined by titrimetry. Twenty-nine trace elements and Br^−^ and I^−^ were analysed by the ICP-MS ELAN DRC II (sample introduced in liquid form), however, we present only few results in this paper, e.g. boron and strontium.

TOC, DOC, HCO_3_^−^, volumetric gas analysis, CO_2_ and DIC were analysed at Vízkutató Vízkémia Kft. in Budapest, using methods MSZ EN 1484:1998 for TOC, MSZ 448-11:1986 for HCO_3_^−^ and MSZ 448-23:1983 for CO_2_. Bicarbonate concentrations determined by this laboratory mostly differ < 2% from MBFSZ's results. DOC was determined from filtrated samples (0.45 μm membrane filter), DIC was calculated from the sum of HCO_3_^−^ and CO_2_, while CO_2_ as a sum of separated and dissolved gas.

Stable isotopes of oxygen and deuterium in water were measured at the Jožef Stefan Institute (JSI) in Ljubljana using the CO_2_–H_2_O (6 h) and H_2_–H_2_O (2 h) equilibration. The equilibrated gases were measured with dual inlet method on a Finnigan MAT DELTA plus IRMS with an automatic H_2_–H_2_O and CO_2_–H_2_O equilibrator HDOEQ48. CO_2_ (Messer 4.5) and H_2_ (IAEA) gases were used as working standards. Two laboratory reference materials calibrated to VSMOW-SLAP scale were used to normalize the results, as well as additional one for control measurements (Vreča, 2016).

Tritium activity, stable isotopes of the dissolved sulphate (for *δ*^34^S_CD_ and *δ*^18^O_SMOW_) and noble gases were measured by the ICER in Debrecen. The tritium concentration of the water samples was determined by the ^3^He-ingrowth method with a VG5400 and a Helix SFT noble gas mass spectrometer using a special isotope dilution technique (Palcsu et al., 2010). The detection limit for tritium is generally 0.02 TU (Papp et al., 2012). Sulphate isotope analyses were done on BaSO_4_ form precipitated from the water. The measurements were taken with a Thermo Finnigan DELTA^PLUS^ XP stable isotope ratio mass spectrometer in continuous flow operation (CF-IRMS). The BaSO_4_ was converted either by an Isolink Flash EA (Thermo Scientific) to SO_2_ or by a high temperature EA to CO to determine sulphur and oxygen isotope ratios. The measurement error of the *δ*^34^S_CD_ and *δ*^18^O_SMOW_ results is ± 0.5 and ± 0.2‰, respectively. Noble gas analysis was done in ICER, Debrecen, Hungary (Papp et al., 2012). Their concentration was measured in four gas samples, having a standard deviation of ± 0.05 ppm for He, ± 0.010 ppm for Ne, ± 5 ppm for Ar, ± 0.005 ppm for Kr, ± 0.5 ppm for Xe and ± 0.015 for R/Ra. In the only water sample (Kraljevi vrelec), the error sums to ± 2E−08 ccSTP/g for He, ± 4E−09 ccSTP/g for Ne, ± 1E−05 ccSTP/g for Ar, ± 1E−09 ccSTP/g for Kr, ± 1E−10 ccSTP/g for Xe, and ± 0.015 for R/Ra.

The *δ*^13^C_PDB_ was measured by ICER on the Thermo Finnigan DELTA^PLUS^ XP with accuracy of ± 0.1‰. The ^14^C measurement was taken at the accredited Hydrosys Labor in Budapest by the laboratory method based on ASTM D6866-06 standard. The CO_2_ gas was extracted by acid admission, from the BaCO_3_ and converted to lithium carbide by absorption onto molten lithium on 500–600 °C. On cooling, the addition of water caused the production of acetylene which was cyclotrimerized to benzene using a vanadium-based catalyst. The radiocarbon activity of benzene was counted by super-low-level liquid scintillation analyser (PerkinElmer Tri-Carb 3170TR/SL). ^14^C values are expressed in pMC (per cent modern carbon).

The ^87^Sr/^86^Sr and *δ*^11^B were determined at the Department of Geology and Geophysics laboratory of the University of Utah. Their fractions were purified using inorganic chromatography and run in a multicollector ICP-MS (Neptune Plus). The quality of the data was checked using Standard Reference Material 987 (National Institute of Standards and Technology) and in-house standard MLR, in turned checked repeatedly against IAEA B-1 and ERM-AE120, ERM-AE121 and ERM-AE122 materials. The values are reported as unique numbers, with information on accuracy of the reference materials. SRM 987 for ^87^Sr/^86^Sr showed 0.71030 ± 0.00001 and MLR for *δ*^11^B had −12.9 ± 0.1.

### Data interpretation

Data analysis and classification were performed in AquaChem 5.1 (Waterloo Hydrogeologic Inc., Canada) with PHREEQC module, in MS Office Excel and HNC-Plot (Karakuş & Aydin, 2017).

Groundwater residence time calculation is a major challenge in reservoirs where deep degassing or water–rock–gas interactions occur along the flow paths. We evaluated the potential effects on the apparent age (*t*) calculations by several equations (as reported in Clark & Fritz, 1997; Friedlander et al., 1981; Ingerson & Pearson, 1964; Szőcs et al., 2013; Trček & Leis, 2017):

1. Uncorrected radioactive decay equation
  1 $$t_{{{\text{uncorr}}}} = 8267 \cdot {\text{ln}}\frac{{{}_{{}}^{14} C}}{{{}_{{}}^{14} {\text{Cs}}}}.$$
2. Chemical correction with bicarbonate concentration as in Trček and Leis (2017)
  2 $$t_{{{\text{corrHCO}}_{{3}} }} = 8267 \cdot {\text{ln}}\frac{{{}_{{}}^{14} {\text{Cg}} \cdot q_{{{\text{tot}}}} {}_{{}}^{14} {\text{Cs}}}}{{{}_{{}}^{14} {\text{Cs}}}}.$$
3. *δ*^13^C correction
  3 $$t_{{{\text{corr}}13C}} = 8267 \cdot {\text{ln}}\frac{{\left( {\delta {}_{{}}^{13} {\text{Cs}} - \delta {}_{{}}^{13} {\text{Cc}}} \right) \cdot {}_{{}}^{14} {\text{Cg}}}}{{\left( {\delta {}_{{}}^{13} {\text{Cg}} - \delta {}_{{}}^{13} {\text{Cc}}} \right) \cdot {}_{{}}^{14} {\text{Cs}}}}.$$
4. *δ*^13^C correction for mantle CO_2_ ver.1
  4 $$t_{{{\text{corrCO}}_{{2}} {\text{\_V1}}}} = 8267 \cdot {\text{ln}}\frac{{\left( {\delta {}_{{}}^{13} {\text{Cs}} - \delta {}_{{}}^{13} {\text{Cc}}} \right) \cdot {}_{{}}^{14} {\text{Cg}}}}{{\left( {\delta {}_{{}}^{13} {\text{C}}._{{{\text{CO}}_{{2}} }} g - \delta {}_{{}}^{13} {\text{Cc}}} \right) \cdot {}_{{}}^{14} {\text{Cs}}}}.$$
5. *δ*^13^C correction for mantle CO_2_ ver.2
  5 $$t_{{{\text{corrCO2\_V2}}}} = 8267 \cdot {\text{ln}}\frac{{\left( {\delta {}_{{}}^{13} {\text{Cs}} \cdot \left( {1 - \frac{{M_{\% } }}{100}} \right) + \delta {}_{{}}^{13} C_{{{\text{CO2}}}} g \cdot \left( {\frac{{M_{\% } }}{100}} \right) - \delta {}_{{}}^{13} {\text{Cc}}} \right) \cdot {}_{{}}^{14} Cg \cdot \left( {1 - \frac{{M_{\% } }}{100}} \right)}}{{\left( {\delta {}_{{}}^{13} C_{{{\text{CO2}}}} g - \delta {}_{{}}^{13} {\text{Cc}}} \right) \cdot {}_{{}}^{14} Cs}}.$$

Two values were used *δ*^13^C_CO2_ = −3.5 and −6.1‰ (Bräuer et al., 2016) in Eqs. 4 and 5. Difference in results is up to 3.3% where heavier carbon–CO_2_ isotope gives higher estimated age.

where

*δ*^13^C_s_: δ^13^C value of DIC in groundwater sample in permil.

*δ*^13^C_c_: δ ^13^C of carbonates; calculations were done with mean 2.2‰ (Koceli et al., 2013).

*δ*^13^C_g_: δ ^13^C of the soil gas CO_2_; calculations were done with –25‰

*δ*^13^C_CO2_g: δ ^13^C of the mantle gas CO_2_; calculations were done with –3.5‰

^14^C_g_:^14^C activity of the soil gas CO_2_ in pMC; calculations were done with 100 pMC

^14^C: initial ^14^C activity in pMC at time of recharge; calculations shown in figures were done with an initial activity value of 60 pMC.

^14^C_s_: ^14^C activity of groundwater sample in pMC.

q_tot_ :  the dilution factor that accounts for carbonate dissolution and inflow of mantle CO_2_. It is calculated dividing the bicarbonate concentration in the recharge area (304 mg/l instead of 200 mg/l as originally used by Pezdič (1997)) with that of the sample and multiplied by assigned factor q (being 0.5 in Trček and Leis (2017).

M_%_: is the mantle gas contribution in%.

To account for high uncertainty in several parameters, we took the Monte Carlo approach. The presented equations had the following parameters varied, all having assigned Gauss distribution:

- Sample bicarbonate concentrations between a given value and ± 10%
- q between 0.7 ± 0.2
- *δ*^13^C_s_ between a given value and ± 0.1‰
- ^14^C_s_ between given a value and ± 0.5 pMC
- *δ*^13^C_c_ between 2.2 and ± 1.0‰

## Results and discussion

Unique chemical properties of mineral water in Rogaška Slatina are well known (e.g. Lapanje, 2006; Nosan, 1973; Ozim, 1978) and we summarize in this paper only the areas and peculiarities observed now. While other wells have been studied as part of regional research by Trček et al. (2011), data on well K-2/75 are published here for the first time.

### Field parameters and hydrogeochemical type of water

Waters from seven wells within this research had temperature from 12.9 to 52.0 °C, pH from 6.3 to 6.9, conductivity (EC) from 4,980 to 11,080 μS/cm and redox potential from −216 to −75 mV.

**Fig. 3:**
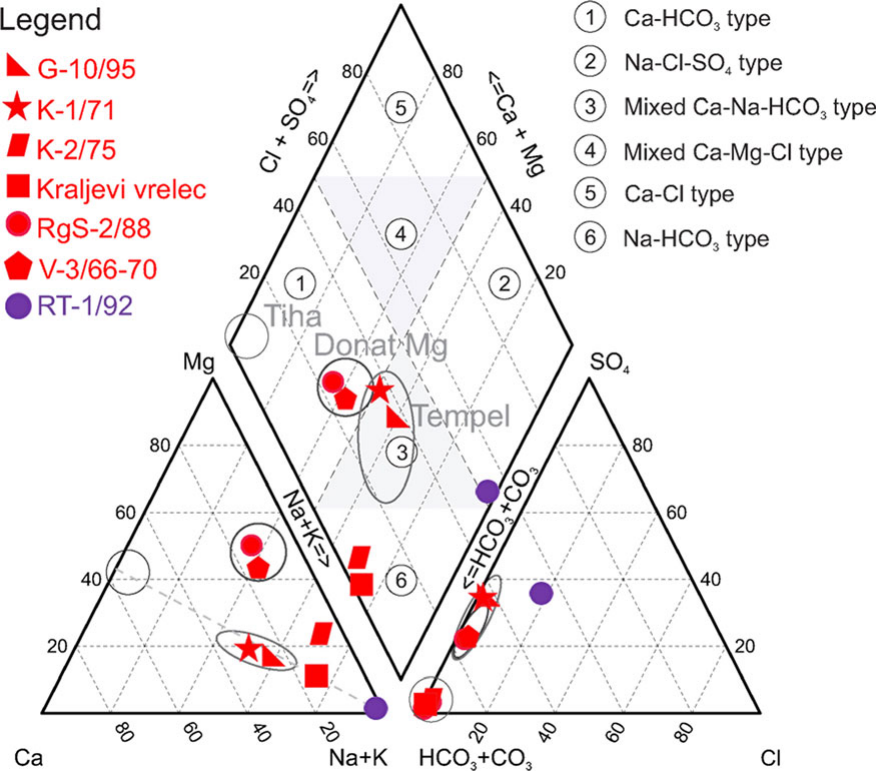
Piper diagram

We determined five water types (Fig. 3):

- Na–HCO_3_: Kraljevi vrelec
- Na–HCO_3_–SO_4_: Rt-1/92
- Na–Ca–HCO_3_–SO_4_: K-1/71, G-10/95
- Na–Mg–HCO_3_: K-2/75
- Mg–Na–HCO_3_–SO_4_: RgS-2/88, V-3/66-70.

The Piper plot (Fig. 3) shows that cation exchange is significant in the non-andesitic aquifers with the end-member in Rt-1/92, however, enrichment of magnesium and sulphate distinguishes the main mineral water wells (RgS-2/88, V-3/66-70) from other.

### Comparison of mineral waters to fresh groundwaters in Slovenia

The investigated waters have very high sodium and chloride concentrations compared to Slovenian fresh groundwaters. Concentrations in this study range from 1050 to 2360 mg/l (average is 1539 mg/l) for sodium and from 26 to 430 mg/l (average is 120 mg/l) for chloride. The order of chloride concentrations increases from Kraljevi vrelec and K-1/71 to G-10/95 to V-3/66-70 and RgS-2/88, followed by K-2/75 and, finally, Rt-1/92. The highest chloride concentrations are associated with higher abundance of clastic rocks. Mezga (2014) describes common values for sodium on average 3.7 mg/l with a median 2.0 of mg/l and a maximum of 36.0 mg/l, while chloride has values of 4.5, 2.3 and 36.7 mg/l. Lapanje (2006) lists that Slovenian thermal and thermomineral waters ever measured (excluding Rogaška Slatina) have a maximum of 8214 mg/l sodium and 12,113 mg/l chloride.

Calcium concentrations in Slovenian fresh groundwaters are on average 60 mg/l, with median 58 mg/l and maximum 152 mg/l, while magnesium has values of 13, 9.5 and 42.0 mg/l, respectively (Mezga, 2014). Thermomineral waters (Lapanje, 2006) have reported a maximum of 640 mg/l calcium and 258 mg/l magnesium. Sampled waters are also enriched. The lowest Ca and Mg concentration (29 and 10 mg/l, respectively, in Rt-1/92) fits into the discussed range and is measured only in the deepest well where cation exchange has finished (Fig. 3). All other waters are enriched in calcium (175–603 mg/l, average 416 mg/l) and magnesium (97–1120 mg/l, average 497 mg/l). Three groups are delineated also on Piper plot (Fig. 3): (a) Kraljevi vrelec and K-2/75, (b) two main mineral water wells (V-3/66-70, RgS-2/88) and (c) K-1/71 and G-10/95. As Ca is increasing in this sequence, Mg deviates so that K-2/75 (423 mg/l) has about half of highest concentrations as denoted in the second group (894–1120 mg/l).

**Fig. 4:**
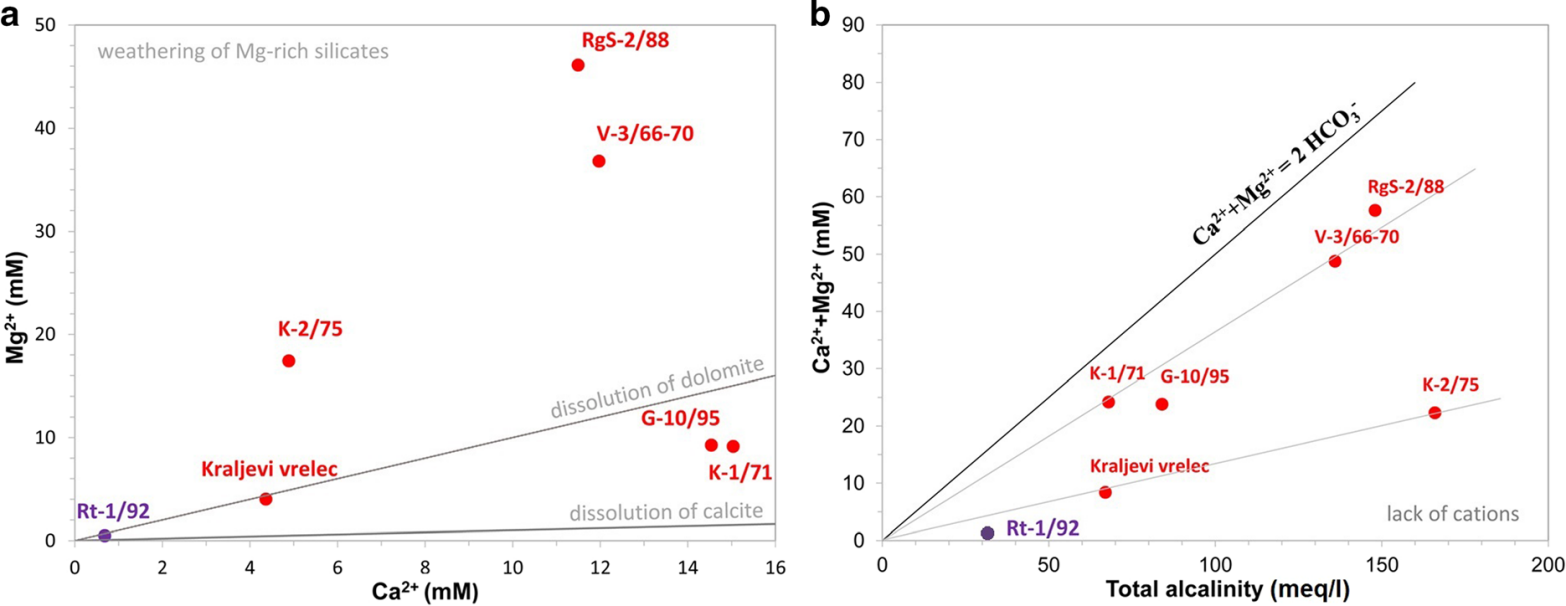
Plots of Ca^2+^ versus Mg^2+^concentrations, denoting dissolution lines of three minerals (**a**), and of Ca^2+^ + Mg^2+^ versus total alkalinity with 1:2 line indicating weathering of dolomites (**b**)

Ca–Mg molar ratio of 164 Slovenian fresh groundwaters was determined to have a median of 3.1 and a minimum of 1.0 (Mezga, 2014). All waters within this paper have ratio below 2. Two groups have values of 1.1 (Kraljevi vrelec) and 1.6 (K-1/71, G-10/95 and Rt-1/92) highlighting dolomite and, to a lesser extent, limestone weathering. The third group with 0.3 (V-3/66-70, RgS-2/88, K-2/75) is evidently different and shows dissolution of Mg-rich silicates (Fig. 4a).

Bicarbonate in Slovenian groundwaters has similar average and median, both being about 235 mg/l, while the maximum is 575 mg/l (Mezga, 2014). The maximum in thermomineral waters was 8052 mg/l (Lapanje, 2006). Our seven waters are similar, having concentrations between 1949 mg/l and 8840 mg/l (Table 2). Sample sequence in the same as for total alkalinity (Fig. 4b) where two main mineral water producing wells have the highest concentrations.

**Table 2:** Main characteristics and general chemical composition of investigated wells

Well name	Temp	EC	pH	Redox	m-Alkalinity	Total hardness	Na^+^	K^+^	Ca^2+^	Mg^2+^	NH_4_^+^
	°C	µS/cm		mV	mmol HCl/l	mg CaO/l	mg/l	mg/l	mg/l	mg/l	mg/l
K−2/75	20.1	10,600	6.8	−214	166	1260	2360	11.0	196	423	2.8
Kraljevi vrelec	12.9	4980	6.3	−202	67	471	1050	17.2	175	97	7.2
V-3/66-70	28.3	10,660	6.7	−143	136	2750	1530	12.2	480	894	0.6
RgS-2/88	15.8	11,080	6.9	−75	148	3240	1470	20.3	461	1120	1.2
G-10/95	17.2	8690	6.6	−216	84	1340	1580	30.9	583	225	4.6
Rt-1/92*	52.0	5730	6.8	189	32***	ND	1600	43.0	29	10	3.3
K-1/71	13.2	7200	6.3	−148	68	1360	1180	30.1	603	222	2.9

Well name	Fe	Mn	Cl^−^	SO_4_^2−^	HCO_3_^−^	SiO_2_	B	Sr	CO_2_	DIC
	mg/l	mg/l	mg/l	mg/l	mg/l	mg/l	µg/l	µg/l	mg/l	mg/l
K−2/75	4.0	0.313	146	175	8840	64.4	4191	10,444	742,000	204,000
Kraljevi vrelec	10.7	0.079	26	1.1	3930	20.9	2412	22,017	1560	1200
V-3/66-70	6.8	0.134	68	2240	7810	115.4	3198	8950	1350	1820
RgS-2/88	3.6	0.090	99	2120	8300	64.4	6160	9060	ND	ND
G-10/95	5.3	0.349	40	2200	4720	50.0	1210	14,872	17,300	5680
Rt-1/92*	0.3	0.010	430	1100	1949	113.0	13,000**	490	1560	ND
K-1/71	10.4	0.486	30	1950	3920	56.3	1175	13,934	ND	ND

All CO_3_^2−^, OH^−^, NO_3_^−^ and NO_2_^−^—concentrations were below 0.1 mg/l. All TOC and DOC concentrations were below 0.5 mg/l. CO_2_ is a sum of separated and dissolved gas. ND = not determined

*These measurements were taken within the regular annual monitoring of water for reporting according to the water concession requirements

**The concentration is questionable as some more recent analyses resulted in only app. 2.5 mg/l

***Alkalinity was calculated from bicarbonate concentrations

Alkalinity between 67 and 166 mmol HCl/l (Fig. 4b, Table 2) is much elevated in comparison with fresh groundwaters in dolomite aquifers in Slovenia (Verbovšek & Kanduč, 2016). It is attributed to enhanced weathering carbonates and evaporites (e.g. sulphate) in the presence of geogenic CO_2_ gas (see Chapter Separated and dissolved cases).

Chemical weathering of rocks increases alkalinity of waters which is enhanced also by CO_2_ degassing in Rogaška Slatina. We have correlated alkalinity, Na^+^, K^+^, Ca^2^^+^, Mg^2^^+^, Cl^−^, SO_4_^2^^−^, SiO_2_, Sr and B concentrations for statistical significance (*p* < 0.05). We found that alkalinity is correlated to magnesium (*r* = 0.77) and boron (*r* = 0.79), and the two among themselves also (*r* = 0.77). Chloride is significantly correlated only to strontium (*r* = −0.83), and strontium to silica (*r* =  −0.88). Besides, calcium is correlated to sulphate (*r* = 0.79). The chemical weathering of rocks (CDW according to Liotta et al., 2016) should produce the ratio between total alkalinity and sum of major cation equivalents to be 1. Deviations are evident in Rogaška Slatina, showing excess of cations in most cases (Rt -1/92 = 2.3, K-1/71 = 1.5, G-10/95 = 1.4, V-3/66-70 = RgS-2/88 = 1.2) that are not derived from CDW. However, depletion in cations is evident at Kraljevi vrelec and K-2/75 (0.9). Both indicate also other geochemical processes. The ratio remains constant when carbonates precipitate, which we can interpret at least at Kraljevi vrelec and K-1/71. If isochemical dissolution of glass matrix occurs, the Mg/(Na + K + Ca) ratio is higher in groundwater than in the glass matrix and the bulk deposition (as taken from basalts at Mt. Etna after Liotta et al., (2016, Fig. 5)). In Rogaška Slatina, such excess in magnesium is calculated at V-3/66-70 and RgS-2/88 (> 0.8), also K-2/75 is close (0.3). Other samples are depleted in magnesium (< 0.2) and fall within the bulk deposition, except for Rt-1/92 with the lowest values (0.01).

**Fig. 5:**
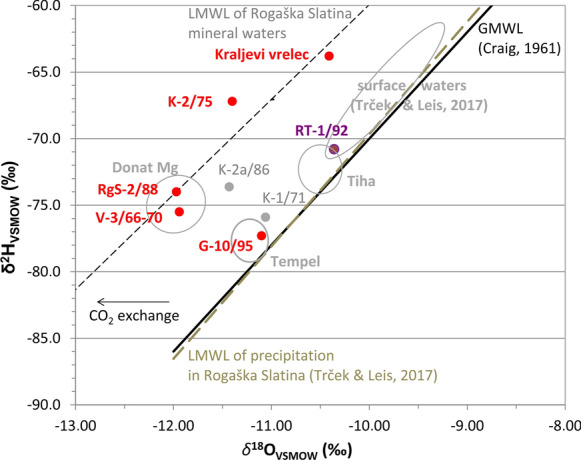
Stable isotopes of waters in Rogaška Slatina. Results for K-2a/86 are taken from unpublished research performed in 2013 while grey dots and spherical areas denote results as published in Trček and Leis (2017)

Total hardness equals carbonate hardness and was measured in waters from six wells, excluding the deepest well (Table 2). The range from 471 to 3240 mg/l CaO gives an average of 1737 mg/l, where two mineral water wells have the highest concentrations. These are much above the average fresh groundwaters, having 56 mg/l CaO (being the same as median) and maximum 151 mg/l (Mezga, 2014).

Iron and manganese are elevated, which was also noticeable during sampling (Fig. 2). Iron concentrations range from 0.3 to 10.7 mg/l (average 5.9 mg/l). Manganese is less abundant and ranges from 10 to 486 µg/l (average 209 µg/l). Usually, the average iron concentrations in Slovenian fresh groundwaters are 36 µg/l, the median 28 µg/l and the maximum 132 µg/l, and 0.82 µg/l, 0.23 µg/l and 22.11 µg/l for manganese (Mezga, 2014).

Ammonium is between 0.6 and 7.2 mg/l with an average of 3.2 mg/l. It is extremely rarely detectable in unpolluted freshwaters in Slovenia (Mezga, 2014). Only one thermomineral water at a hydrocarbon research site has 59 mg/l (Lapanje, 2006).

Silica ranges from 20.9 to 115.4 mg/l (average 69.2 mg/l). As expected, it is highest in andesitic aquifer and the deepest well, and the lowest in the shallowest well Kraljevi vrelec. These concentrations are much above the range of Slovenian fresh groundwaters (Mezga, 2014) where the average of 6.8 mg/l, the median of 4.0 mg/l and the maximum of 28.8 mg/l are reported. Very few thermomineral waters have silica above 50 mg/l, the highest having 147 mg/l (Lapanje, 2006).

Many parameters (EC, Na^+^, NH_4_^+^, Fe, B, often also pH, Mn, SO_4_^2−^ and Cl^−^) significantly exceed drinking waters standards (Drinking Water Directive, 1998) and several confirm anoxic conditions in the aquifers.

### Saturation indices

All investigated waters are saturated to oversaturated with dolomite, calcite, calcedony, quartz and siderite (Table 3). Only Rt-1/92 is slightly unsaturated with aragonite. All are undersaturated with anhydrite and gypsum. V-3/66-70, RgS-2/88 and Rt-1/92 are oversaturated with goethite and hematite, and the first two also with talc. K-2/75 is saturated with hematite and talc, but not with goethite.

**Table 3:** Saturation indices for characteristic minerals. Tolerant equilibrium range was taken as ± 0.1 SI for calcite, ± 0.5 SI for dolomite (López-Chicano et al., 2001) and ± 0.1 SI for other minerals

Station ID	Aragonite	Calcite	Dolomite	Chalcedony	Quartz	Siderite	Goethite	Hematite	Anhydrite	Gypsum	Talc
K-2/75	0.8	0.9	2.5	0.7	1.1	0.9	−1.0	0.0	−2.0	−1.8	−0.1
Kraljevi vrelec	0.0	0.2	0.2	0.3	0.7	0.7	−2.1	−2.2	−3.9	−3.7	−7.4
V-3/66-70	1.0	1.2	3.0	0.8	1.2	1.1	0.8	3.6	−0.7	−0.5	2.0
RgS-2/88	1.1	1.2	3.1	0.7	1.2	0.9	1.4	4.8	−0.7	−0.5	1.1
G-10/95	0.7	0.9	1.6	0.6	1.0	0.7	−1.5	−1.0	−0.4	−0.2	−3.0
Rt-1/92	−0.2	0.0	−0.1	0.5	0.9	−0.1	6.6	15.3	−1.6	−1.5	−0.7
K-1/71	0.4	0.5	0.8	0.7	1.2	0.6	−1.2	−0.4	−0.4	−0.1	−5.0

These results align with observed carbonate scaling (Fig. 2) and discussed processes in following chapters.

### Water origin by stable isotopes

#### Oxygen and deuterium in water

This research provided new data on isotopic composition of K-2/75.

Stable isotopes in water vary from  −11.97 to  −10.30‰ for *δ*^18^O and from  −77.3 to  −63.8 for *δ*^2^H (Table 4) which is in the range of previous publications (Brenčič & Vreča, 2006; Pezdič, 1997; Trček & Leis, 2017).

**Table 4:** Isotopic composition of mineral waters. Because of high gas/water ratio the fractionation effects on carbon isotopes related to CO_2_–water interaction are not relevant

Well name	*δ*^18^O_H20_	*δ* ^2^H_H20_	*d*-excess	^3^H	*δ* ^13^C_DIC_	^14^C	*δ* ^34^S_CD_	*δ* ^18^O_SMOW_	*δ* ^11^B	^87^Sr/^86^Sr
	‰	‰		TU	‰	pmC	‰	‰	‰	
K-2/75	−11.40 ± 0.00	−67.2 ± 0.0	24.0	0.000 ± 0.019	1.28	2.05 ± 0.20	27.7	10.5	31.7	0.70813****
Kraljevi vrelec	−10.41 ± 0.01	−63.8 ± 0.4	19.5	2.991 ± 0.117	−1.78	1.16 ± 0.12	ND	ND	28.4	0.71060
V-3/66-70	−11.94 ± 0.02	−75.5 ± 0.5	20.0	0.000 ± 0.019	1.33	1.03 ± 0.10	27.4	10.1	12.5	0.70708
RgS-2/88	−11.97 ± 0.02	−74.0 ± 0.6	21.8	0.035 ± 0.026	0.99	1.52 ± 0.15	26.6	8.9	16.4	0.70699
G-10/95	−11.05 ± 0.03	−77.3 ± 0.5	11.5	0.011 ± 0.022	−0.86	5.16 ± 0.48	28.0	11.1	26.4	0.70824
Rt-1/92*	−10.30 ± 0.00	−69.9 ± 0.1	12.5	< 0.2	−4.37**	1.43**	ND	ND	11.3***	0.71060***
K-1/71	−11.06 ± 0.01	−75.9 ± 0.6	12.6	ND	ND	ND	ND	ND	ND	ND

*Is explained in Table 1

**The results are taken from Trček and Leis (2017)

***Water for B and Sr isotopes was sampled on 6.09.2016 for the needs of presented research

****An additional (duplicate) sample of K-2/75 had a value of 0.70810

We agree with previous interpretations on local meteoric origin of waters (Pezdič, 1997; Trček & Leis, 2017) and identification of CO_2_ degassing (Clark & Fritz, 1997) at two end-members (Brenčič & Vreča, 2014; Trček & Leis, 2017): mineral water from andesitic aquifer (V-3/6-70, RgS-2/88) and Kraljevi vrelec, where freshwater mixes with naturally outflowing mineral water. We supplement these data with additional sample K-2/75 which is positioned in between (Fig. 5). It indicates warmer climate during infiltration in comparison with waters in V-3/66-70 and RgS-2/88. In comparison with the local meteoric water line (Trček & Leis, 2017), oxygen shift of these three waters is estimated to about −1.3‰. CO_2_ mineral springs in the NE part of Slovenia have oxygen shift up to 0.6‰, −1.8‰ was measured in a 0.5 km deep thermomineral well in Radenci, and about −3‰ at a mofette Stavešinske Slepice (Rman N., unpublished). This oxygen depletion indicates that flux of CO_2_ gas is sufficiently high to modify the oxygen isotope composition in Rogaška Slatina.

On the other side, Brenčič and Vreča (2014) investigated *d*-excess in bottled waters and calculated it to be 17.4–18.6‰ in Donat Mg water. In contrast to Pezdič (1997), they argued that increased *d*-excess is a consequence of the water–CO_2_ interaction. We also do not assign these values to orographic precipitation, as described, e.g. in Liotta et al. (2006), because precipitation and surface waters (Trček & Leis, 2017) already do not indicate this. If we apply the calculation, *d*-excess would be 20.0–21.8‰ for two Donat Mg wells and 19.5 and 24.0‰ for its mixtures (Table 4); however, there the dominant process is oxygen shift and not *d*-excess. Other groundwaters have *d*-excess in the range of 11.5–12.6‰ (Table 4), close to the results of Brenčič and Vreča (2014) for the Edina and Tempel brands (12.5–13.1‰), while their Tiha had only 10.9‰. These waters are obviously not included in our survey. Such values are comparable to fresh groundwaters in Slovenian dolomites (11.0–16.9‰), which are mostly recharged with precipitation from the western part of the Mediterranean basin (Verbovšek & Kanduč, 2016).

The stable isotopes in our mineral water end-member are not as depleted as typical Pleistocene groundwaters in the Pannonian basin which were recharged during cold periods, but are more similar to interglacial waters in the Pontian-Pliocene formation with a noble gas recharge temperature (NGT) of 6 °C or thermomineral waters in the metamorphic basement (Szőcs et al., 2013). On the opposite side, Rt-1/92 is closer to waters with NGT = 15 °C, implying warm infiltration. Groundwaters stored in Pliocene clastic rocks in Velenje (Kanduč et al., 2014) cover the whole range of our stable isotope values, but their dating has not been done so no comparison is possible.

#### Carbon in water

All dissolved (DOC) and total organic carbon (TOC) concentrations are below 0.5 mg/l. Dissolved inorganic carbon (DIC) is a sum of CO_2_ and bicarbonate ions, and because gas was not measured in RgS-2/88 it could not be calculated. DIC is very high, between 1200 and 204,000 mg/l.

**Fig. 6:**
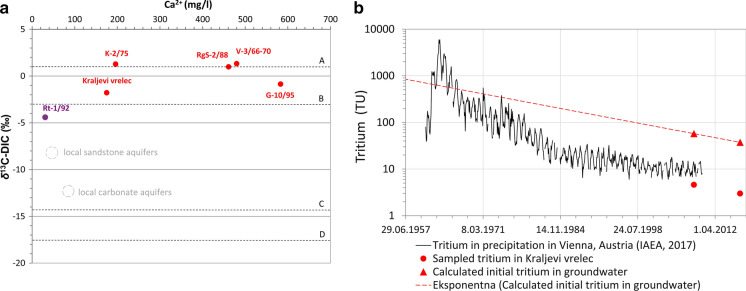
Carbon isotopes in dissolved inorganic carbon (DIC) for sampled waters (δ^13^C_DIC_) where Rt-1/92 and grey circles denote previous information from Pezdič (1997) and Trček and Leis (2017). Four processes are marked (Kanduč et al., 2014): (A) dissolution of carbonate with average composition 1.2‰; (B) dissolution of carbonates with −2‰ as resulting in water with −3‰; C) disequilibrium dissolution of carbonate (+ 1.2‰) with carbon acid from soil CO_2_ (−26.6‰) with value −14.2‰; D) open system equilibration of DIC with soil CO_2_ from degradation of organic matter (**a**); Tritium curve fitting for Kraljevi vrelec well. The Vienna precipitation values are taken from IAEA WISER database. Dashed line represents the exponential curve of the radioactive decay of tritium. This curve is crossing the Vienna tritium time series at the calendar years of 1960–1965 (**b**)

Stable carbon isotopes were analysed in five waters (Table 4). *δ*^13^C between −1.78 and + 1.33‰ (Fig. 6) is a result of host carbonate rock dissolution (Mazor, 1997). Brenčič and Vreča (2007) reported slight enrichment in heavy isotopes in bottled Donat Mg water (*δ*^13^C = 0.5–1.2‰) and similar stands also for Trček and Leis (*δ*^13^C = 0.53–2.05‰; 2017). The latter data were also used for Rt-1/92 as none our analyses were performed.

Koceli et al. (2013) reported *δ*^13^C in carbonate rocks in Slovenia to be from −2.0 to + 4.1‰, with average 2.2‰, which is also a typical value for Triassic dolomites near Rogaška Slatina. Mezga (2014) calculated the average *δ*^13^C in Slovenian groundwaters to be −12.1‰, median −12.9‰ and maximum −0.7‰. The latter is characteristic for carbonate aquifers.

Significant methane contribution might affect the *δ*^13^C of DIC mainly if *δ*^13^C_CH4_ is strongly depleted, having lower values than −30‰ (Palcsu et al., 2014). In our research, methane was detected only at K-2/75 and Kraljevi vrelec with a maximum of 0.3 vol% in separated gas (Table 6). Very low concentrations were reported also previously (see Chapter Separated and dissolved cases), so we estimate that its effect on DIC is negligible.

A hypothesis on dissolution of marine carbonate rocks as noticed by previous publications (Brenčič & Vreča, 2006; Pezdič, 1997; Trček & Leis, 2017) can again be accepted. Moreover, we noticed also that enrichment in *δ*^13^C_DIC_ appears not only due to carbonate dissolution but especially due to mantle degassing as it rises with higher mantle gas contribution (R/Rac) with a clear positive linear relationship (*r* = 0.94).

### Estimation of mean residence time

#### Tritium in water

Because low tritium activity was reported in mineral waters (Pezdič, 1997; Trček & Leis, 2017) we performed analyses with very low detection limit. The exception was Rt-1/92 where we took available data.

Most investigated water can be considered as free of tritium and only one, Kraljevi vrelec with depth of 24 m, had 2.991 ± 0.117 TU (Table 4). In July 2008, this water had 4.6 TU (Trček & Leis, 2017). We have assumed a binary mixing model where tritium-free mineral water is mixed with tritium active freshwater component and their shares are constant in time. Based on oxygen-18 mass balance we estimated there is app. 8% of freshwater. Both tritium values are positioned on the decay curve and were corrected for the dissolution. This resulted in freshwater activity at the time of sampling of 57.5 TU in 2008 and 37.4 TU in 2016. The latter value is exactly the result of the radioactive decay of the earlier value. This allows us to calculate the tritium concentration back in time and see how it fits to the tritium time series of the Vienna precipitation. As can be seen in Fig. 6, most probably the fresh component was recharged between 1960 and 1965, so app. 50 years prior to 2016. This fits within the mean residence time estimated at deeper freshwater spring waters in the region which were dated to 30–60 years in 2008 (Trček & Leis, 2017). Two flow systems mix in Kraljevi vrelec: local circulation of freshwater with mean residence time of slightly above 50 years and regional regime of deep tritium-free mineral water, which here naturally outflows to the surface. Dating of this deep end-member is explained in chapter on radioactive carbon.

Better time constrain for the fresh end-member could usually be achieved by the tritium–helium dating method (Palcsu et al., 2017) but, unfortunately, it is not applicable here because of huge mantle contribution of helium (Bräuer et al. (2016) and this paper).

For identified freshwater component we evaluated the vertical recharge rate based on a simple analogue from the Great Hungarian Plan where tritium peak was followed in depth profiles (Palcsu et al., 2017). Simple linear calculation was done assuming that 50-year-old bomb-peak freshwater reached the bottom of our 24 m deep well. This gives a recharge rate of approximately 48 mm/year which is in the range of clastic sediments in the Pannonian basin (Palcsu et al., 2017). Similar recharge values, of about 45 mm/year, have been estimated in previous studies based on tritium measurements from soil moisture profiles, base-flow measurements of streams and hydrogeochemical modelling (Horváth et al., 1997; Szőcs, 2005; Tóth et al., 1997). These calculations foresee that vertical flow is predominant, and, in our experience, less than 10% of such infiltration into the shallower aquifer infiltrates into the deeper ones. In our case, the average annual precipitation is 1100 mm/y (source Atlas Okolja—https://gis.arso.gov.si/atlasokolja) which would give a rough vertical infiltration of only 5%. This means that the Oligo-Miocene siltstone covering the mineral water aquifer has very low vertical permeability and most of groundwater discharges along the shallow flow paths.

#### Carbon-14 in water

Radiocarbon was measured in water from five wells. Its concentration ranges between 1.03 ± 0.10 and 2.05 ± 0.20 pMC with an outlier of 5.16 ± 0.48 pMC (Table 4) which supports the hypothesis of Pleistocene age recharge. Concentrations decrease from G-10/95, K-2/75, RgS-2/88 and Rt-1/92 to Kraljevi vrelec and V-3/66-70.

Archive data from Trček and Leis (2017) were taken for comparison and we noticed that their values differ from ours for both, *δ*^13^C and radiocarbon. In the first case, our *δ*^13^C is depleted for 0.7–2.6‰. In the second case, their radiocarbon concentrations are lower than our lowest ones (accounting for standard deviation) in three waters and unexpectedly higher in one (G-10/95, + 3.37 pMC). If the difference is not caused by an error, it might imply depletion of the mineral water aquifer with the old end-member especially closer to the recharge area. But no shift is yet evident in the stable isotope plot (Fig. 5). It might be worth a trial to use the radiocarbon analyses for annual monitoring for several years and then see whether the freshwater intrusions really exist.

Kraljevi vrelec water is again highlighted as a mixture of two waters: the fresh one contributes active tritium and enrichment in heavy stable isotopes of water while the mineral water imprints CO_2_–water exchange in oxygen-18, almost positive carbon-13 and very low radiocarbon concentration. We interpret this with fast natural outflow of mineral water at Kraljevi vrelec. It is interesting that the other well with freshwater intrusions, K-2/75, has almost double the radiocarbon concentration even though it is much deeper. We assume that this and high chloride concentration result from water from the overlying Oligo-Miocene bituminous mica marlstone.

Interpretation of mean residence time of these mineral waters is very challenging because of strong inflow of geogenic CO_2_ gas that enhances carbonates dissolution and silicate hydrolysis (Trček & Leis, 2017), as expected (Carreira et al., 2008; Clark & Fritz, 1997; Suckow, 2014). Previous apparent age calculations were published with rather definite values, but we took the Monte Carlo approach instead. Initial radiocarbon concentrations and dilution factors have high uncertainty of determination, so we rather pointed out that there is no simple solution to the age determination and any applied method has some systematic differences to others. Some equations gave even negative solutions which are represented by 0 (Table 5).

**Table 5:** Calculated retention time of mineral waters using Eqs. 1–5 (see Chapter Methodology) and with P90, P50 and P10

Percentile	Equation 1	Equation 2	Equation 3	Equation 4	Equation 5	Previous apparent age*
Rt-1/92						
P90	27,800	11,800	20,000	33,200	31,600	
P50	30,900	16,800	23,300	36,300	34,700	14,000
P10	35,700	22,400	28,300	41,100	39,400	
V-3/66-70						
P90	29,000	2000	0	0	3600	
P50	33,400	7900	9200	22,000	7800	7100
P10	40,700	15,600	19,300	31,200	15,000	
RgS-2/88						
P90	27,500	0	0	0	13,800	
P50	30,400	4400	9000	21,800	16,700	3400
P10	34,800	9500	16,000	27,800	21,100	
K-2/75						
P90	25,600	0	0	0	13,600	
P50	27,900	1400	4200	17,000	15,900	–
P10	31,000	5600	11,800	23,500	19,000	
G-10/95						
P90	19,300	0	2400	16,800	10,900	
P50	20,300	0	6500	19,300	11,900	7200
P10	21,400	1900	9200	21,000	13,000	

* Published by Trček and Leis (2017) using Eq. 2. Zero denotes negative results

**Fig. 7:**
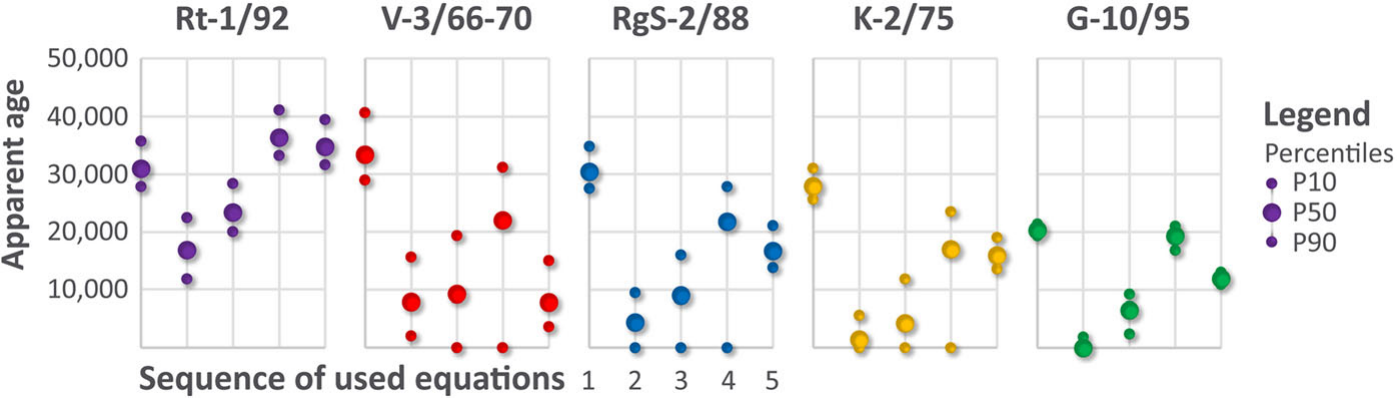
Exemplified percentiles of calculated mean residence times for five thermal and mineral waters by 5 equations (Eqs. 1–5) accounting for various effects of carbonate dissolution and inflow of mantle CO_2_

Differences in apparent groundwater ages vary within a few 10,000 years, with the maximum of 41,000 years (Fig. 7) and such large age window illustrates high uncertainties. Overestimation is usually provided by the uncorrected radioactive decay equation (Eq. 1) as it does not account for observed carbonate dissolution. Consequently, Eq. (1) is not applicable for interpretation. The youngest times are calculated by chemical correction with bicarbonate concentration as in Trček and Leis (2017; Eq. 2). In our case (using concentrations from 2016), this approach often results in negative ages, so we assume that it cannot be applied here with high reliability. Unfortunately, also the enrichment in *δ*^13^C_DIC_ due to mantle degassing rather limits the applicability of the *δ*^13^C correction method (Eq. 3) for waters in the studied area.

Two correction methods are based on the *δ*^13^C value of mantle CO_2_ gas (Bräuer et al., 2016) where version 1 (Eq. 4) used only concentrations and isotopic ratios while version 2 (Eq. 5) also the shares of mantle gas contribution. Generally, Eq. 4 gave slightly higher apparent ages than Eq. 5 but its P10 was negative in all three wells with observed CO_2_ degassing on stable isotope plot so we may assume that its applicability can be rather limited sometimes also for our case. Equation 5 seems to give reasonable value but high apparent ages closer to the recharge area are not hydrogeologically feasible.

**Fig. 8:**
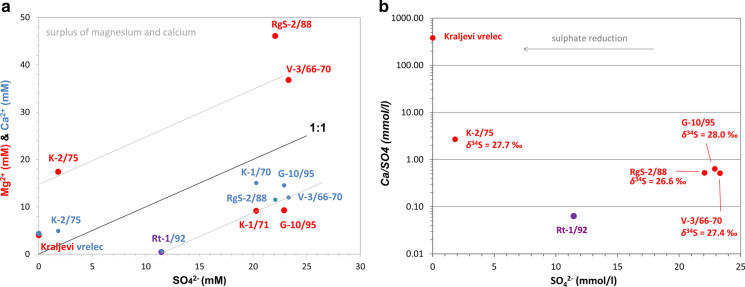
Calcium, magnesium and sulphate concentrations (**a**) and ratios (**b**) in investigated waters

From this comparison, we conclude that the mean residence times, in general, do increase along the flow path (Fig. 8), from G-10/95 closest to the recharge area to the deepest well Rt-1/92. Water in K-2/75 is slightly younger than in V-3/66-70 and RgS-2/88. It is very hard to differentiate between the latter two waters if uncertainties in initial parameters are accounted for, as we did. RgS-2/88 is expected to have shorter mean residence time as the well is shallower, but at the same time, this is a natural outflow zone so the oldest end-members should emerge jointly.

Stable isotopes of the mineral water end-member show that the water was recharged in a colder climate than today, but do not match the very light isotopic signal of the typical LGM. Mixed waters and Rt-1/92 most likely recharged at warm conditions. Because there were several successions of glacial and interglacial events during the Pleistocene and uncertainly in apparent age determination is high, we cannot specify the exact timing of recharge. The waters appear to have been recharged during a period that can be investigated only by radiocarbon dating methods (Aggraval et al., 2009), but even these do not provide a clear result.

### Origin of sulphur, boron and strontium in water

#### Sulphur in water

With exception of Kraljevi vrelec having only 1.1 mg/l, other six waters are enriched in sulphate, having it of 175–2240 mg/l (Table 2). Concentrations lower than 2 g/l occur in clastic aquifers (Rt-1/92) or due to mixing with fresh groundwaters from clastic rocks (K-1/71, K-2/75). Molar Ca/SO_4_ ratio is not typical for gypsum dissolution or sea water (Clark & Fritz, 1997), and it is 381 at Kraljevi vrelec, 2.7 at K-2/75, 0.5–0.7 at V-3/66-70, RgS-2/88, G-10/95 and K-1/71, and 0.06 at Rt-1/92 (Fig. 8). Possibility of sulphate reduction in Kraljevi vrelec and Rt-1/92 water could not be argued because sulphate concentrations at Kraljevi vrelec were too low to determine its isotopes while Rt-1/92 was not investigated. Sulphate concentration in fresh groundwaters in Slovenia is much lower (Mezga, 2014) with an average of 10.0 mg/l, median 5.7 mg/l and maximum of 67.4 mg/l. Moreover, Verbovšek and Kanduč (2016) report the range from 1.7 mg/l to 97 mg/l in waters from dolomite aquifers.

Two hypotheses were set regarding the sulphate origin: (a) oxidation of pyrite and (b) dissolution of evaporites in carbonate rocks from Boč Mt. The investigated waters have very similar isotope ratios and are enriched in heavy sulphur isotopes—*δ*^34^S from 26.6 to 28.9‰ (average 27.4‰) and *δ*^18^O from 8.9 to 11.1‰ (average 10.2‰; Table 4, Fig. 8), denoting only one origin of sulphate. As both values are strongly positive, oxidation of pyrite is excluded and dissolution of evaporite minerals in Triassic carbonate rocks is a predominant origin of sulphur.

Values are significantly enriched in heavy isotope of sulphur in comparison with Slovenian rivers, lakes and tap waters, and also in comparison with the evaporitic sulphates and structurally substituted sulphates of the Karavanke Mountains and in western Slovenia rocks (*δ*^34^S = 12–24‰; Dolenc (unpublished in Vokal-Nemec et al., 2006)). Fórizs et al. (2019) analysed thermal waters in Budapest karst with *δ*^34^S = 9.7–17.7‰ and *δ*^18^O = 4.2–5.4‰. They attributed sulphate to dissolution of Permian evaporites with an average *δ*^34^S = 12.8‰ as Triassic evaporites have higher values, from 16.0 to 33.0‰ (average 24.3‰). Values are close to ocean water with 21‰ (Clark & Fritz, 1997) and fit even better to the Lower to Middle evaporite sulphate (gypsum/anhydrite) values during the so-called Röt event with *δ*^34^S 27‰ instead of the usual 12–17‰. However, its *δ*^18^O remained closer to ordinary values between 10 and 18‰ than being 16‰ as at the Röt event (Claypool et al., 1980) so different Triassic carbonate rocks with evaporites were dissolved.

Observed lower values of oxygen isotope can also be a consequence of oxygen exchange with water. As water temperature is mostly below 30 °C, more than 18.500 years are needed to observe such effect (Fórizs et al., 2019). The mean residence time of mineral waters is long and still highly debatable, estimated from 3400–14,000 years (Trček & Leis, 2017) to much more (this paper).

Bacterial sulphate reduction (noticeable by bacteria or H_2_S) may result in lower measured values of both isotopes than the original according to Fórizs et al. (2019); however, we are more in favour of hypothesis of Onac et al. (2011) which states that bacterial sulphate reduction results in depleted H_2_S isotopic composition and enriched remaining sulphate in water. Observed pyrite in volcanoclastic rocks might even be a result of this process as there is lots of iron available in the mineral water but only litter organic matter (see first chapters). Some sulphate reducing bacteria (*Thermodesulfobacterium* and *Desulfotomaculatum*) were determined in water from V-3/66-70 and RgS-2/88 (Börger, 2017), yet they are not predominant species in these waters. No smell of H_2_S was noticeable at any of the locations but its actual concentration was not analysed and, moreover, no analysis of sulphate isotopes of aquifer rocks is known to the authors. This is important for further studies of its origin as Nakano et al. (2020) attributed values 24.0–28.9‰ of two bottled waters in Japan to volcanic origin because some volcanic hot springs have *δ*^34^S = 20–30‰.

#### Boron in water

**Fig. 9:**
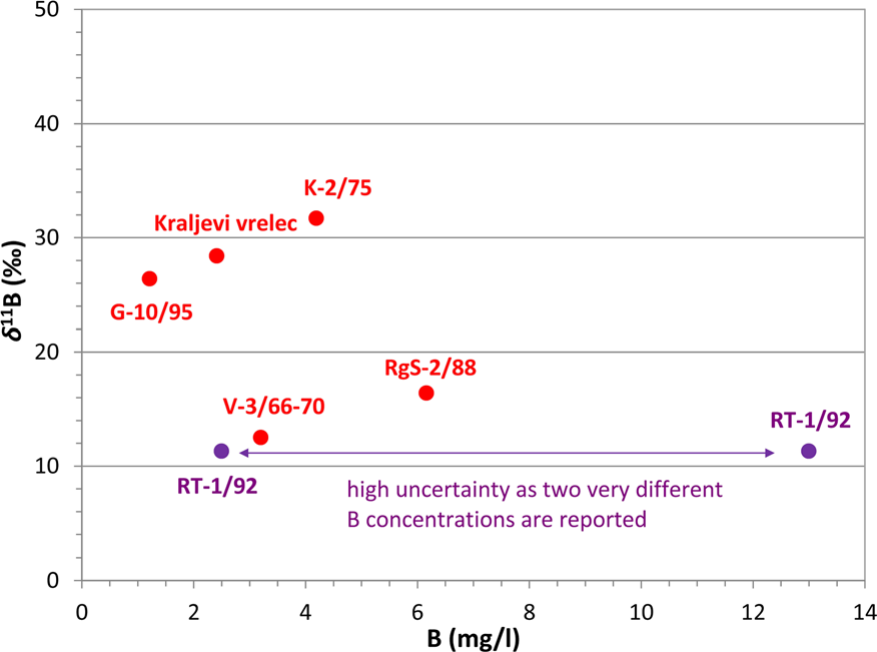
Boron concentration and δ^11^B isotopes in water

Boron concentrations in waters from six wells varied from 1.2 to 6.1 mg/l, giving an average of 3.1 mg/l. If only Donat Mg wells are used, 4.5 mg/l is calculated (Fig. 9, Table 2). An additional sample at Rt-1/92 with 13 mg/l has questionable accuracy. Boron isotopes were determined in six wells, *δ*^11^B ranging from 11.3 to 31.7‰ (Table 4).

Molar B/Cl ratios from 0.10 (G-10/95, K-2/75, Rt-1/92), 0.13–0.20 (RgS-2/88, V-3/66-70, K-1/71) to 0.31 (Kraljevi vrelec) show enrichment in boron in comparison with sea, ground and hydrothermal waters (Vengosh et al., 1998). Considering various known mixing processes at wells, we may conclude that ratios are close to typical values for weathering of andesitic rocks (Trompetter et al., 1999), which is here the main mineral water aquifer. Basalts of Mt. Etna have molar Mg/B and Na/B ratios of about 1700 and 740 (Liotta et al., 2016). Our investigated waters have ratios of 2–124 and 112–614, respectively. Enrichment in boron point out that chemical weathering of, in this example, basaltic matrix is not the only source of boron.

However, these groups are not the same as evident from isotopes where only two groups appear: depleted in heavy isotopes with *δ*^11^B = 11.3–16.4‰ (RgS-2/88, Rt-1/92, V-3/66-70), and enriched in heavy isotopes of boron with *δ*^11^B = 26.6–31.7‰ (other wells).

Four hypotheses on boron origin are: a) magmatic/mantle origin along with other gases, b) dissolution of andesitic rocks, c) hydrothermal alterations, and d) dissolution of marine rocks. The first one is rejected as the isotopic composition should be much more depleted than is observed in our case (Lū et al., 2014; Nisi et al., 2014; Vengosh et al., 1998). The second hypothesis is also rejected as Purnomo et al. (2016) and Vengosh et al. (2002) point out that dissolution of igneous and andesitic rocks produces values of 0‰, which we do not observe. Third hypothesis is feasible. Values below 9.3‰ (Vengosh et al., 1998) or at about 13‰ (Lū et al., 2014) can be attributed to (hydro)thermal waters, so our deepest wells could have been exposed to some hydrothermal alterations which may occur based on the tectonic evolution of the region. Our higher isotopic values are slightly depleted in comparison with sea water with 39.6‰ (Purnomo et al., 2016), whereas Williams et al. (2001) give range for formation sea waters between 31 and 43‰. Farber et al. (2004) support the hypothesis that values around 30‰ result from carbonate dissolution although we cannot rule out gypsum contribution with similar value. Therefore, the fourth hypothesis on dissolution on marine carbonates can be accepted for wells G-10/95, K-2/75 and Kraljevi vrelec.

### Strontium in water

We used non-conservative strontium (Sr) to distinguish among aquifer rocks and identify mixing of waters as is enters dissolution–precipitation processes of carbonate, sulphate and clay minerals (Calligaris et al., 2018; Voerkelius et al., 2010). Dolomite minerals reduce strontium concentration in comparison with dissolution of pure limestones but dolomitization does not affect the isotopic ratios (Faure et al., 1978). Its isotopic equilibrium with aquifer rock (Frost & Toner, 2004) is reached as Rogaška Slatina waters have sufficiently long mean residence time.

**Fig. 10:**
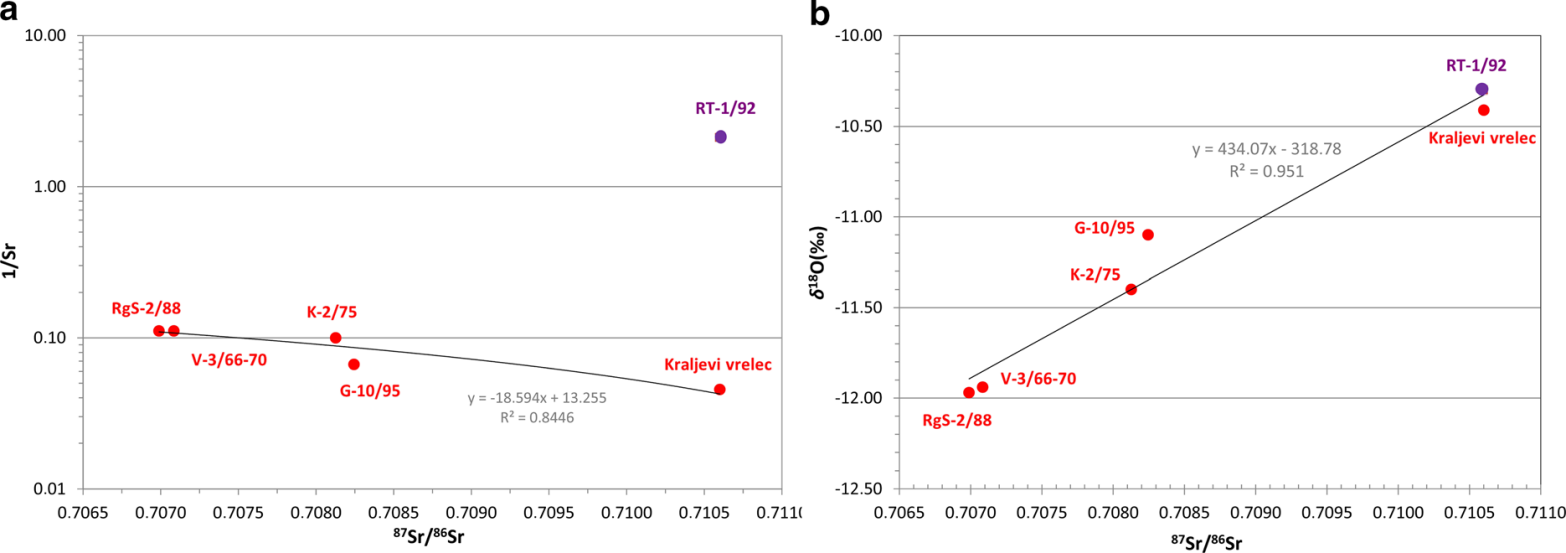
Concentration and isotope ratio of strontium (**a**) with comparison to oxygen isotope in water (**b**)

**Fig. 11:**
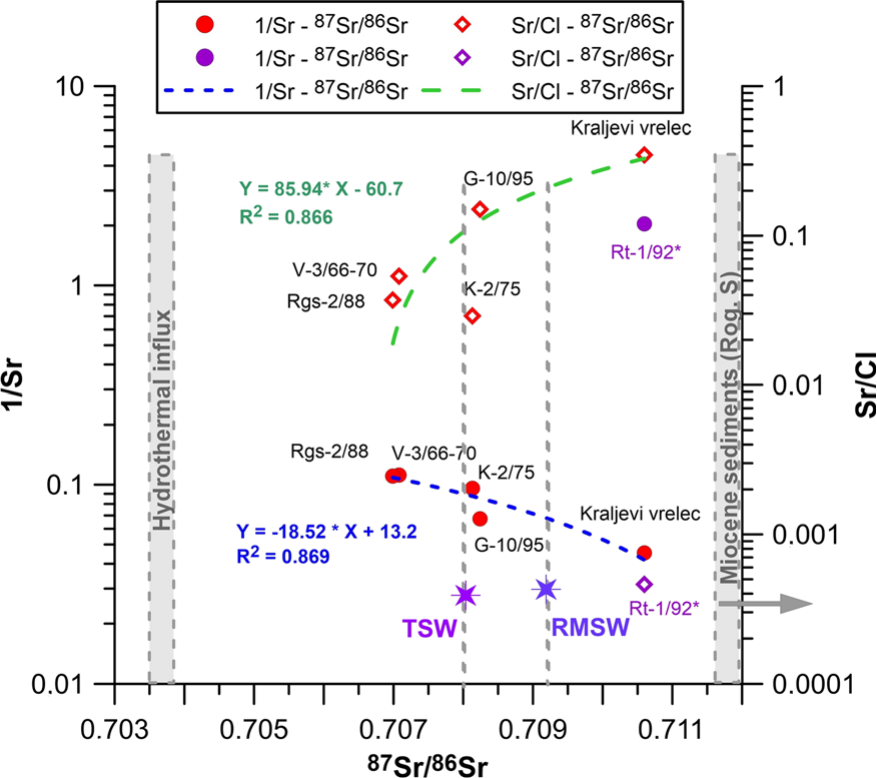
Concentration and isotope ratio of strontium (left *y*-axis) and comparison to Sr/Cl concentration (right *y*-axis). RMSW—Recent Mediterranean Seawater (Henderson et al., 1994; Liotta et al., 2017); TSW—Triassic Seawater (Kovács et al., 2020); Hydrothermal influx (Pearce et al., 2015; Spooner, 1976); Miocene sediments Rogaska Slatina (Kocsis et al., 2008)

Strontium concentrations in the seven waters varied from 0.5 to 22.0 mg/l, where the end-members are the deepest and the shallowest well, and an average of 11.4 mg/l (Table 2). Obviously, strontium is more soluble in cold waters (Table 2). Strontium isotopes were analysed in water from six wells, ^87^Sr/^/86^Sr ranging from 0.70699 to 0.71060, with an average of 0.70861 (Figs. 10, 11, Table 4).

Four groups were identified: a) highest Sr concentration (22.0 mg/l) with high ^87^Sr/^/86^Sr = 0.7106 isotope ratio, b) high Sr concentrations (10–15 mg/l) with ^87^Sr/^/86^Sr at about 0.7082, c) moderate Sr concentrations (9.0 mg/l) with ^87^Sr/^/86^Sr at about 0.7070, and d) lowest extreme in Sr concentration (0.5 mg/l) with ^87^Sr/^/86^Sr = 0.7106. Beside the original porewater and/or meteoric water, the hypotheses on strontium origin are related to dissolution of various aquifer rocks: (i) Triassic carbonate rocks, (ii) Oligocene andesitic rocks, and iii) Miocene clastic rocks.

Since only one reference (Kocsis et al., 2008) has been found regarding Sr isotope data of aquifers in the study region and very diverse values are reported worldwide, depending on age and lithology of aquifers, our Sr data interpretation has some limitations and uncertainties. Regarding comparable volcanic rocks—andesites, waters from Devonian andesites in England have Sr ratio of 0.70660 (Montgomery et al., 2006), from Miocene–Pliocene andesites in Los Azufres 0.7049 and from Long Valley caldera riolites 0.07078–0.7080 (Pinti et al., 2013). The Upper Triassic carbonates from Italy had ^87^Sr/^/86^Sr ratios between 0.70776 and 0.70791 (Faure et al., 1978). Vuataz et al. (1988) noticed hot spring in volcanics with 0.708–0.710 where higher values of 0.715–0.722 were measured at outflow path to Paleozoic carbonates and shales. Natural mineral waters in Europe show values 0.7035–0.7070 if they are from Tertiary and Quaternary basalts, and higher values of 0.7090–0.7110 if from clastic rocks with grains of older rocks (Voerkelius et al., 2010). Waters from carbonates in N Portugal are reported to have app. 0.709485 (Carreira et al., 2008). In the Pleistocene–Upper Miocene siliciclastic sediments of the Great Pannonian Plain in Hungary, Sr ratio in ground and thermal waters rises along the flow path, being between 0.709 and 0.712 (Szőcs et al., 2015). Groundwater from basalt rocks of Mt. Etna, Sicily, has Sr ratio of 0.7032–0.7039, from Cretaceous to Miocene sedimentary rocks 0.707 and more and from Oligo-Miocene flysch up to 0.7178 (Liotta et al., 2017). The Miocene tectonism and volcanism has strongly affected the study region and surroundings. The Mediterranean Sea and the Parathetis had different levels of connectivity with the Indian and Atlantic Ocean in the Miocene affecting their water composition during time. Mediterranean Sea water is characterized by an average 0.7092 value (Henderson et al., 1994; Liotta et al., 2017).

In Slovenia, very localized information is available. Miocene (upper and middle–upper Badenian) sediments west of Rogaska Slatina (at Mestinje) have been investigated (Kocsis et al., 2008) which show 0.7118–0.7165 values in sediments and 0.7088 in fossils. In the Classical Karst Region, Calligaris et al. (2018) noticed that the Sr ratio gradually decreases from the river water (0.70837–0.70847) which infiltrates and dissolves Cretaceous limestone to a typical karst water with 0.70750. In Bled, five springs and freshwater wells from Triassic carbonate rocks have the ^87^Sr/^/86^Sr ratio 0.70838 ± 0.00018 on average, while four thermal waters from Upper Pannonian and Pontian Mura Formation siliciclastic sandstone in NE Slovenia have it 0.71310 ± 0.00119 (Rman, unpublished data). Investigation of eight Slovenian bottled waters (Zuliani et al., 2020) fits within these values. Radenska is in accordance with water from the Upper Pannonian and Pontian siliciclastic sandstone. Six waters from predominantly Triassic carbonate rocks have an average of 0.70927 ± 0.001448, pointing out carbonate weathering. Most radiogenic water is produced from Voda 902 where carbonate aquifer is influenced also by clastic rocks. The ratio of 0.71942 ± 0.00026 is a result of silicate weathering. Moreover, groundwaters from a coal mine in Velenje were investigated; outflowing from the Pliocene clastic aquifer have Sr ratio of 0.70820–0.71056, while from the Triassic carbonates 0.70808–0.70910 (Kanduč et al., 2016).

In Rogaška Slatina, a simple linear mixing relationship is indicated in two plots (Fig. 10). 1/Sr–^87^Sr/^/86^Sr has a strong negative correlation (*r* = −0.92) where higher Sr concentrations result also in higher isotope ratio. Deepest, thermomineral water (Rt-1/92) is an exception as its Sr concentration is the lowest and does not fit the line. *δ*^18^O–Sr ratio plot has a strong positive correlation (*r* = 0.98), where higher Sr isotope ratios correlate with enrichment in heavy oxygen isotope in water. We also compared molar ratios of chloride and strontium to seawater and sedimentary brine from Mt. Etna to identify mixing (Liotta et al., 2017) but no such resemblance was found. Nevertheless, linear relation between Sr and Cl is evident, starting with Sr-richest and Cl-poorest water from Kraljevi vrelec, and shifting over G-10/95, V-3/66-70, RgS-2/88 and K-2/75 to Sr-poorest and Cl-richest thermomineral water from Rt-1/92 well. Sr/Cl versus ^87^Sr/^/86^Sr gives the same sequence of waters as the 1/Sr–^87^Sr/^/86^Sr plot.

We calculated mixing ratios of two end-members based on simple mass balance equations; RgS-2/88 from andesitic aquifer and Rt-1/92 from clastic one. V-3/66-70 holds 98% of water from in RgS-2/88, which we believe is within the measurement error of both parameters. K-2/75 has between 32 and 34% of RgS-2/88 water, and K-1/71 approximately 54% (only based on oxygen because Sr ratio was not determined). G-10/95 deviates from the trend line (Fig. 10) and oxygen-18 denotes 55% of water from RgS-2/88 while Sr ratio only 35%. The reason is that this well does not show a signature of water–CO_2_ isotopic exchange (Fig. 5) as others, therefore, it is not directly comparable to the set and Sr probably reflects more realistic ratio. Shallowest well Kraljevi vrelec has the same Sr ratio as Rt-1/92 while based on oxygen-18 93% of water is similar as to RgS-2/88.

From the presented information we conclude that Sr depleted in heavy isotopes, forming the third (c) group, originates from andesitic rocks (Montgomery et al., 2006; Pinti et al., 2013; Voerkelius et al., 2010). It is characteristically less radiogenic than the Slovenian groundwaters from carbonate aquifers but slightly more radiogenic than, for example, the Italian bottled water from volcanic aquifer (Zuliani et al., 2020). It is also just at the upper boundary of groundwaters from volcanoclastic Mt. Etna rocks and, at the same time, the on lower boundary of sedimentary basement rocks (Liotta et al., 2017), obviously having strontium isotopes the least affected by sedimentary rocks. Its Sr isotopic composition most likely reflects the hydrothermal influx characterized by more unradiogenic values (^87^Sr/^86^Sr ~ 0.70366) (Pearce et al., 2015). Spooner (1976) highlights ~ 0.7039 value is required as ^87^Sr/^86^Sr ratio of the oceanic crust to maintain the current strontium isotopic composition of seawater by isotopic exchange during hydrothermal convection within spreading oceanic ridges. The noble gas data support a very strong mantle connection with about 87.2–97.2% mantle He origin. The predominant Sr origin in the second, mixed (b) group with higher Sr concentrations and ratios is attributed to a strong dissolution of Triassic carbonates, with isotopic values very similar to Triassic carbonates and Triassic Sea water (Kovács et al., 2020). Observed values are close to published ones (Calligaris et al., 2018; Faure et al., 1978; Zuliani et al., 2020). Sr ratio does not increase along the flow path as in intergranular terrestrial sedimentary sequences (Szőcs et al., 2015) as these wells are closer to the recharge area and main aquifer is formed by younger andesitic rocks. In the first (a) group with highest Sr isotopic values and concentrations, we can say that the ratio increases along the flow path. This increase in isotopic ratio at Kraljevi vrelec can most likely be attributed to input from continental Miocene sediments with higher radiogenic Sr isotope and mixing with fresh groundwaters from clastic rocks. The mixing with fresh groundwater is also supported by its tritium (2.991 ± 0.117 TU) content which reflects a groundwater component younger than 60 years. The lowest Sr concentration (group d) in the deepest well Rt-1/92 can be a result of its removal by ion exchange and carbonate precipitation (Fig. 3). It has similarly high values of Sr isotope ratio as group a). According to noble gas data, it is least affected by mantle connection, therefore its original Sr isotope ratio is not likely to be affected by hydrothermal influx. This well is hosted in Triassic non-carbonate but clastic rocks, which explains enrichment in heavier isotopes. Based on these assumptions, we can conclude that the sources of similarly enriched Sr isotope ratios in Kraljevi vrelec and Rt-1/92 are different.

### Composition and origin of gases

#### Separated and dissolved cases

Whole set of gas analyses was performed at three wells but information on gas composition is available also for V-3/66-70. Two most interesting mineral water wells could not have gas flow measurements done but their most resembling one is K-2/75. H_2_S was not monitored.

Total gas–water ratio is very high, from 862 l/m^3^ at Kraljevi vrelec to 424,777 l/m^3^ at K-2/75 (Table 6). Gas in wells is predominately free (90.9–99.8%) except for the shallowest Kraljevi vrelec (19.5%) where natural mixing occurs.

**Table 6:** Water and gas flow rates and ratios. Dissolved gas ratios can be calculated from subtracting separated gas from the total

Well name	Water flow rate	Gas flow rate	Total gas–H_2_O ratio	Total CH_4_–H_2_O ratio	Separated gas–H_2_O ratio	Separated CH_4_–H_2_O ratio
	l/min	l/min	l/m^3^	l/m^3^	l/m^3^	l/m^3^
K-2/75	0.25	105.9	424,777	1285	423,933	1285
Kraljevi vrelec	5	0.8	862	0.49	167	0.49
RgS-2/88	2.14	–	–	–	–	–
G-10/95	6.32	55.3	9774	0	8881	0

V-3/66-70, Rt-1/92 and K-1/71 were not sampled

Predominant gas component in both, dissolved and separated gas, is CO_2_. It constitutes 98.84–99.64 vol% of dissolved gas and 95.23–98.76 vol% in separated gas (Table 7).

**Table 7:** Separated and dissolved gas composition of samples, and calculated percentages as being without air or without CO_2_

Well name	Separated gas in the sample				Separated gas without air		Dissolved gas in the sample				Dissolved gas without air			Dissolved gas without CO_2_		
	CO_2_	CH_4_	O_2_	N_2_	CO_2_	CH_4_	CO_2_	CH_4_	O_2_	N_2_	CO_2_	CH_4_	N_2_	CH_4_	O_2_	N_2_
	vol%	vol%	vol%	vol%	vol%	vol%	vol%	vol%	vol%	vol%		vol%	vol%	vol%	vol%	vol%
K-2/75	95.23	0.30	1.50	2.96	99.68	0.32	99.64	0.01	0.11	0.25	99.96	0.01	0.03	3.13	29.35	67.53
Kraljevi vrelec	98.76	0.30	0.22	0.72	99.70	0.30	98.84	0	0.38	0.78	99.99	0	0.01	0	33.04	66.96
V-3/66-70	1.95	0	21.00	77.05	100.00	0	99.41	0	0.17	0.42	99.93	0	0.07	0	70.61	29.39
G-10/95	96.46	0	0.90	2.64	100.00	0	99.49	0	0.17	0.34	100.00	0	0	0	33.61	66.39

At dissolved gas without air, O_2_ is not listed as it is 0 vol% at all samples

Without air, CO_2_ is almost pure (99.93 to 100.00 vol%) and very similar numbers stand for the separated phase (99.68–100.00 vol%).

Methane is detected only in minor amounts at two wells. Kraljevi vrelec has 0.30 vol% only in separated gas while K-2/75 has the same concentration in separated gas and lower (0.01 vol%) in dissolved gases. Bräuer et al. (2016) published 0.005 and 0.011 vol.% of thermogenic methane at V-3/66-70 and RgS-2/88, which is below or just at the detection limit of our measurements and in accordance with the first gas survey ever (Pezdič, 1997).

#### Noble gases

Four of six waters were analysed as a gas phase (Table 8) and the same number of additional wells supplements the information on gases as published in Bräuer et al. (2016).

**Table 8:** Noble gases in gas samples

Well name	He	Ne	Ar	Kr	Xe	R/Ra	^4^He/^20^Ne*	He terr	3He terr	R/Ra terr	**Mantle He**
	ppm	ppm	ppm	ppm	ppb						**%**
K-2/75	2.08	0.026	54.2	0.006	< 0.1	5.37	88.4	2.049592	1.54E−05	5.44	83.6
V-3/66-70	2.17	0.003	1.0	0.008	< 0.1	6.32	799.4	2.169439	1.90E−05	6.32	97.3
RgS-2/88	0.49	0.001	37.5	0.003	0.03	5.47	541.6	0.468961	3.68E−06	5.67	87.2
G-10/95	7.35	0.016	123.8	0.018	1.88	4.85	507.7	7.280545	4.92E−05	4.89	75.2

*Precision of this result is not optimal as it was determined later

All six wells show high helium excess (Tables 8, 9), in fact extremely high ^3^He/^4^He ratios even worldwide (see references in Bräuer et al., 2016) that indicate strong mantle origin of the helium. The sample V-3/66-70 has the highest helium isotope ratio ever found in shallow continental gas in Europe. Water sample Rt-1/92 suffered subsurface degassing. Therefore, their paleo-infiltration temperatures (NGT) could not be calculated which is very unfortunate because it would help to reduce uncertainties in radiocarbon apparent age calculations.

**Table 9:** Noble gases in water samples

Well name	He	Ne	Ar	Kr	Xe	R/Ra	^4^He/^20^Ne**	He terr	3He terr	R/Ra terr	Mantle He
	ccSTP/g	ccSTP/g	ccSTP/g	ccSTP/g	ccSTP/g						%
Kraljevi vrelec	1.16E−06	5.24E−09	2.98E−05	3.08E−09	1.48E−10	4.72	244.7	1.16E−06	7.57E−12	4.73	72.7
Rt-1/92*	4.28E−05	3.35E−07	4.38E−04	7.53E−08	4.81E−09	1.06					16.3

*Is explained in Table 1

**Precision of this result is not optimal as it was determined later

**Fig. 12:**
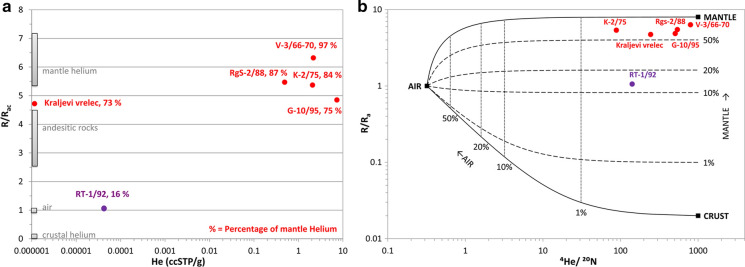
Helium isotopic ratio in the sample (R) and in the air (Ra) versus its concentration (**a**) and versus ^4^He/^20^Ne (**b**; Karakuş & Aydin, 2017) to evaluate the contribution of air, crust and mantle origin

We can distinguish among two groups regarding their content of noble gases. Mineral waters have less than 1% of atmospheric helium and are supplied with 73–97% of helium from the subcontinental lithospheric mantle (SCLM). We used 6.1 ± 0.9 R/Ra as the mantle helium end-member (Gautheron & Moreira, 2002) but several other values are also reported (Fig. 12a), e.g. 6.32 (Gautheron et al., 2005) and 8 (Pinti et al., 2013). From such high gas fluxes as discussed in the previous chapter, we conclude that CO_2_ has the same, mantle origin. The main inflow zone along a fault zone is closest to V-3/66-70. As expected, the lowest (but still extreme) mantle contribution is evident at the shallowest Kraljevi vrelec and at G-10/85, closest to the recharge area.

In comparison with previous measurements (Bräuer et al., 2016), our values are 16–20% higher. If the variation is not a consequence of errors, this might imply reservoir depletion over time where lower reservoir pressure enhances inflow of gases along deep and open Šoštanj Fault Zone.

The deepest well Rt-1/92 still has approximately. 16% of mantle-derived helium and the rest from the crust (Fig. 12b). The crustal contribution is similar as in the Upper Pannonian geothermal aquifer in the Pannonian basin (Szőcs et al., 2013), implying very long mean residence time of groundwater.

## Conclusions

The presented research provided new insights into the origin of the individual dissolved components in mineral waters and their gases. In addition to the standard approach of interpreting the chemical composition and stable isotopes of oxygen, deuterium and carbon in water, we have also used isotopic techniques which are still too rarely used to distinguish between the origins of sulphur, strontium, boron and noble gases. This is quite a novelty in Slovenia and the wider region, especially in the study of mineral and thermal waters. These methods have proven to be very applicable in hydrogeological systems where it is necessary to distinguish between different aquifer lithologies, as in our case. Nevertheless, the interpretation presented would be more accurate if the properties of the host rocks were known. This is a challenge that needs to be addressed in the future so that most of the hypotheses put forward can be verified with local datasets.

The general findings on the meteoric origin of water are consistent with those of previous researchers and indicate strong influx of geogenic gases, helium and CO_2_ from the mantle, which promote the dissolution of carbonate rocks with evaporite minerals as well as clastic and volcanoclastic rocks. High magnesium concentrations in bottled mineral water are found to be predominantly from chemical weathering of andesite rocks, which is not true for boron, which is derived from hydrothermal alteration and dissolution of marine carbonate rocks. Sulphate is predominantly evaporitic, while strontium reflects weathering of all three lithology types mentioned earlier.

Dissolution and mixing processes are not straightforward and not all components react similarly. Binary mixing is sometimes evident, and the deepest well does not respond as the end-member of the entire hydrogeological system of mineral waters. Dating of mineral waters was discussed by the Monte Carlo approach and there is no simple time constraint. Paleo-infiltration temperatures that might help could not be calculated because of subsurface degassing, and no other current dating technique is applicable because we assume that the waters were recharged several 1000–10,000 years ago.

It is obvious that such special natural mineral and medicinal waters were formed due to exclusive local hydrogeological conditions. This is also the reason why more geological, hydrogeological and hydrogeochemical research is needed to reduce the uncertainties shown and to learn more about such systems in the future.

## Data Availability

All used data are presented in tables in the paper.
